# Ultrasound-Responsive Systems as Components for Smart Materials

**DOI:** 10.1021/acs.chemrev.1c00622

**Published:** 2021-11-12

**Authors:** Athanasios G. Athanassiadis, Zhichao Ma, Nicolas Moreno-Gomez, Kai Melde, Eunjin Choi, Rahul Goyal, Peer Fischer

**Affiliations:** † Micro, Nano, and Molecular Systems Group, Max Planck Institute for Intelligent Systems, Heisenbergstrasse 3, 70569 Stuttgart, Germany; ‡ Institute of Physical Chemistry, University of Stuttgart, Pfaffenwaldring 55, 70569 Stuttgart, Germany

## Abstract

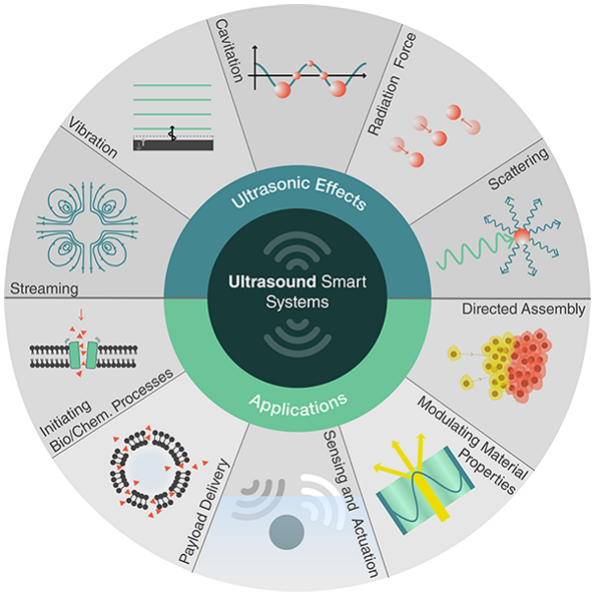

Smart materials can respond to stimuli and adapt their responses based on external cues from their environments. Such behavior requires a way to transport energy efficiently and then convert it for use in applications such as actuation, sensing, or signaling. Ultrasound can carry energy safely and with low losses through complex and opaque media. It can be localized to small regions of space and couple to systems over a wide range of time scales. However, the same characteristics that allow ultrasound to propagate efficiently through materials make it difficult to convert acoustic energy into other useful forms. Recent work across diverse fields has begun to address this challenge, demonstrating ultrasonic effects that provide control over physical and chemical systems with surprisingly high specificity. Here, we review recent progress in ultrasound–matter interactions, focusing on effects that can be incorporated as components in smart materials. These techniques build on fundamental phenomena such as cavitation, microstreaming, scattering, and acoustic radiation forces to enable capabilities such as actuation, sensing, payload delivery, and the initiation of chemical or biological processes. The diversity of emerging techniques holds great promise for a wide range of smart capabilities supported by ultrasound and poses interesting questions for further investigations.

## 1 Introduction

Recently, there has been a growing demand for smart systems or devices that can change between defined states and respond to external stimuli in an adaptive manner. Depending on the complexity, smart systems possess mechanisms or components for sensing, memory storage, computation, energy harvesting, actuation, and communication. At large scales, a smart system can have completely embedded computers that provide some of these functionalities. However, as the length scales of operation decrease below hundreds of microns, it becomes increasingly difficult to build conventional computers and actuators onto a device. Instead, we look to alternative designs where the smart capabilities are coded not into a computer but rather into specific material properties and physical (or chemical) interactions between the components that comprise the system. Biological systems are of course the pinnacle of known smart materials, with all biological functionalities arising from a mixture of physical and chemical interactions with the environment, along with biochemical information that is ultimately encoded in DNA. However, as one looks to develop smart material systems for various scientific and technological endeavors, it can often make sense to break from biological paradigms and exploit different kinds of physical effects to achieve smart functionality.

Traditional physical systems and effects that are used for smart behavior include electric fields, magnetic fields, and light. In these cases, the underlying mechanisms are clear, and there are well-known examples of responsive systems utilizing these effects. For instance, piezoelectric materials can be used to couple mechanical motion or forces to electrical signals for feedback and sensing in smart structures,^1^ magnetorheological fluids can provide external control over fluid behavior such as adhesion,^2^ certain polymers respond to pH and temperature,^3,4^ and photochromic materials can be used to induce coloration in transparent optical materials in the presence of light.^5^

Recently, ultrasound has emerged as an alternative tool to shape and impart functionality to smart materials. Ultrasound can deliver energy remotely for sensing, actuation, or communication, and it provides several qualitative benefits over optical, magnetic, and electrical fields in many contexts. Acoustic fields can propagate through opaque or complex media with low losses, can be localized to small regions in space and time, and can be tuned more than 12 orders of magnitude in frequency to effectively couple with phenomena and objects at different time and length scales. Moreover, ultrasonic sources (frequencies above 20 kHz) and technologies form the cornerstone of many mature industries, such as healthcare and nondestructive testing, making robust sources and techniques available for adaptation to new applications.

Although ultrasound can often complement the optical, magnetic, and electrical effects used in smart systems, the physical mechanisms that can be used to enable smart functionality in ultrasonic systems have long remained unexplored. The availability of miniaturized electronics and precise light emission and detection technologies over the past few decades has led to a dominance of electronic and optical techniques in the development of smart systems. As a result, ultrasound has long been overlooked as an alternative and successful implementation of smart ultrasonic systems has lagged behind the other fields.

Nonetheless, the past few years have seen several innovations that are accelerating the adaptation of ultrasonic components for use in smart systems and materials. This trend has been supported largely by a shift toward integration of smart systems with biological systems, which benefit from the above-mentioned advantages of ultrasound. Recent developments in fields such as drug delivery, energy harvesting, and genetic engineering have identified new systems triggered by ultrasound that can be adapted for use in more general smart systems. In this article, we review recent progress in this field and provide an introduction to the different key ultrasonic techniques, their implementations, and their capabilities.

**Figure 1:**
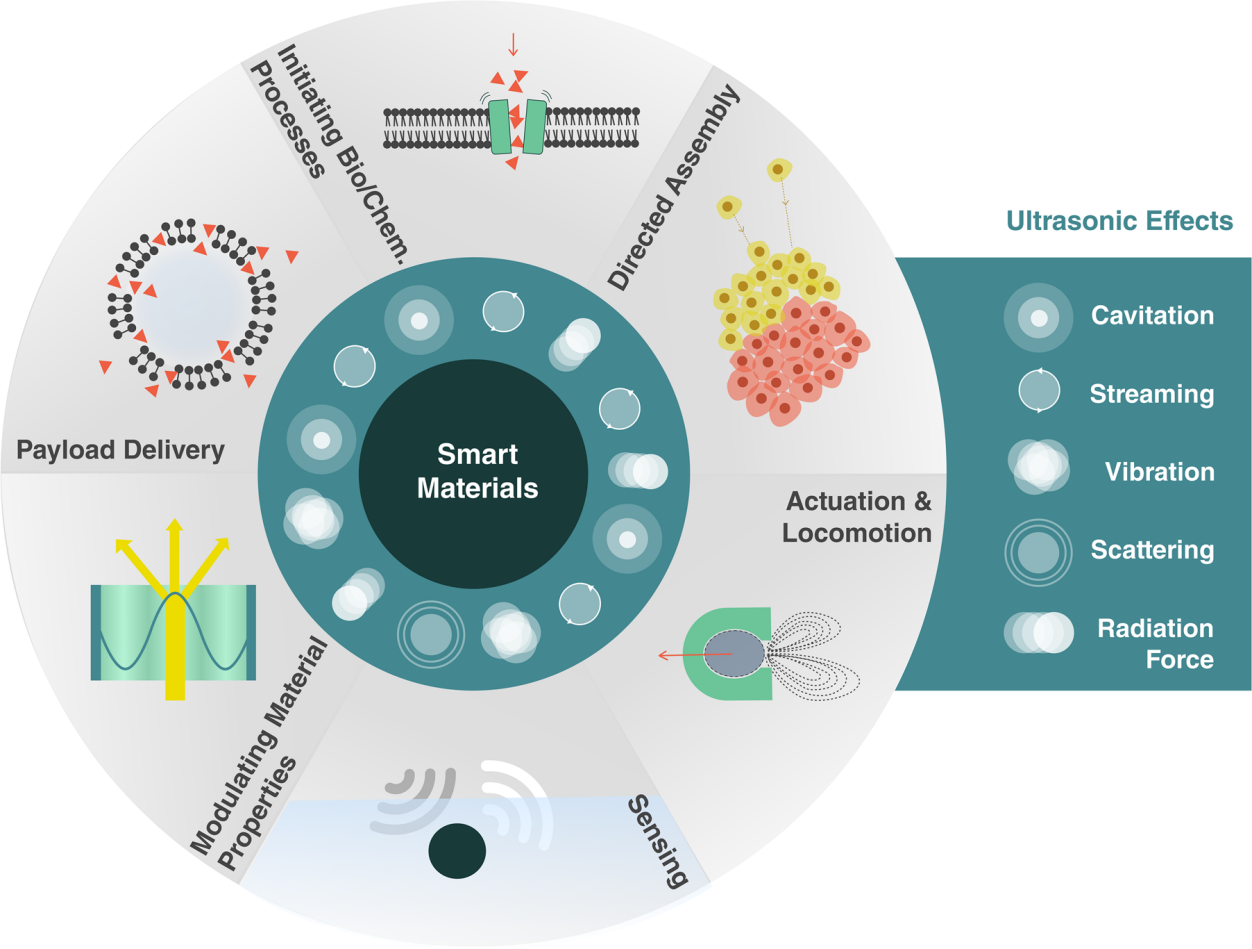
Capabilities of smart materials enabled by ultrasound.

We consider how ultrasound can support six classes of smart capabilities, depicted in Figure 1. These range from directed assembly of smart materials, geometric reconfiguration of smart systems, sensing and actuation, payload transport and delivery, to the triggering of biological and chemical processes. These capabilities are enabled by different ultrasound-induced effects, namely, cavitation, microstreaming, structural vibrations, acoustic scattering, and the acoustic radiation force. This review explores the basis of these effects and how they can be utilized, tuned, and combined with other physical, chemical, and biological systems to enable unique responsive systems and smart material capabilities.

This review complements other recent reviews on subfields of acoustics (e.g., acoustofluidics,^6^ nanoacoustics,^7,8^ particle manipulation,^9,10^ and microbubble acoustics^11^) in both scale and scope. It is not restricted to any particular scale or single mechanism within acoustics. Rather, it aims to bring together all of the recent developments that can be applied in the development of smart and responsive systems. It focuses on the intersection of acoustics with smart systems and not only identifies mechanisms and techniques that could be useful for smart systems, but also explores emerging directions and open questions in this rapidly developing field.

## 2 Background: Acoustics and Ultrasound

### 2.1 What Are Acoustic Waves?

The field of acoustics deals with the transfer of energy through matter via mechanical waves. Generally, an acoustic wave is excited in a medium using a transducer, which converts electrical signals into vibrations that are transferred to the medium. These vibrations propagate through the medium as mechanical waves of compression and expansion. For many applications, it is necessary to understand how these waves will interact with any boundaries or objects that may be present. Ultimately, we want to use these underlying principles to describe and predict how acoustic waves will (1) transport energy or information to a recipient, such as a sensor, actuator or responsive material, and (2) how that information or energy can be converted into other forms of useful work.

**Figure 2:**
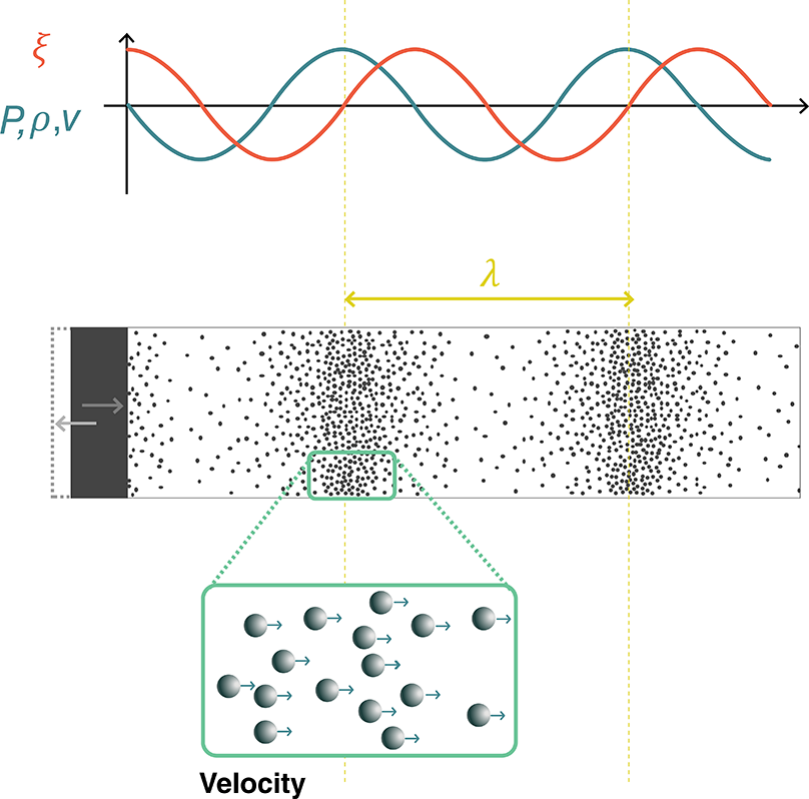
Acoustic waves generated by the motion of a boundary. Acoustic waves are mechanical waves of compression and rarefaction in a medium. Within the wave, the local pressure *p*, density ρ, particle velocity *v*, and particle displacement ξ vary as a function of time and position, with specific relationships between these variables. Density, pressure, and velocity oscillate in phase, while the displacement is 90° out of phase.

Acoustic waves carry energy through compression and microscopic motion in a medium. While the acoustic waves are ultimately reliant on intermolecular interactions, it is easier to consider the wave causing motion of conceptual small bodies or “particles” in the medium, which represent a region much larger than the atoms or molecules but small enough that the wave behavior is effectively constant within. The acoustic wave can then be described continuously through the medium using different acoustic quantities: the *pressurep*, *particle velocityv⃗*, *particle displacement* ξ⃗, and *density fluctuation* ρ. As is seen below, these quantities can ultimately be used interchangeably, depending on the problem at hand. As a wave propagates, the acoustic quantities will vary in time and with the position in the medium, as shown in Figure 2. While it is possible to describe the propagation of a *wave*, it is common to refer to the shape of an *acoustic field*, which means the values of *p*, *v*, ξ, or ρ at every point in space, and usually indicates an interest in spatial patterns associated with the wave.

Acoustic waves are typically excited in a medium by a moving boundary. For instance, ultrasonic transducers use piezoelectric materials to convert electrical energy into mechanical vibrations. This oscillating boundary motion creates regions of high density (compression) and low density (rarefaction) that travel outward from the transducer, as shown in Figure 2. In the compressed regions pressures are higher than ambient pressure, while in the regions of rarefaction they are lower. Since the pressure varies in space, there is a net force in any given region of material, driving the local particle velocity.

An acoustic excitation travels through the material with a finite speed *c*, known as the material’s sound speed. In a fluid, the sound speed depends on the fluid’s equilibrium density ρ_0_ and its adiabatic bulk modulus *K*:^12^
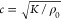
1

In solids, wave propagation is more complicated because, in addition to compressional interactions, elastic solids also support shear interactions. Such shear coupling leads to a large number of different wave types that can propagate in solids, depending on geometry and mechanical properties. In the simplest case of an infinite (or very large) solid body, two types of waves can propagate: bulk compressional (longitudinal) and shear (transverse) waves. Their propagation speeds are given by
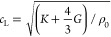
2
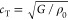
3where *G* is the solid’s shear modulus. When interfaces are present, then additional wave types may propagate, including Rayleigh waves at an interface, flexural waves in a plate, and axial and torsional waves in bars, among others. In many cases, acoustic wave propagation in solids can be approximated considering only compressional wave behavior. This simplification is most accurate when waves propagate at normal incidence through a solid. Below, we will only consider acoustic waves in fluids and ignore elastic wave effects. A complete treatment of elastic waves can be found in standard texts.^13^ As indicated by eq 1, the stronger the bulk modulus (and thus the intermolecular forces), the higher the sound speeds: solids tend to have higher sound speeds than liquids, which in turn tend to have higher sound speeds than gases. (While the density suggests that the lower density of gases would increase the sound speed compared to solids, the bulk modulus changes more and, therefore, has a larger role in setting the sound speed.)

Most often, acoustic systems are driven *harmonically*, or sinusoidally at a single frequency *f*. In this case, *p*, *v⃗*, *ξ⃗*, and ρ will oscillate at the driving frequency in time, forming periodic waves in space with a wavelength λ = *c*/*f*. (This is true for small amplitude waves, but for large amplitudes or when significant nonlinearities are present, additional harmonics could arise.)

While acoustic waves span an enormous range of frequencies from below 1 Hz (atmospheric infrasound) to over 100 GHz (crystal lattice vibrations), this review is mainly concerned with ultrasound in the range 20 kHz–50 MHz. This range is of particular interest because these frequencies (1) fall outside the range of human hearing; (2) interact safely and with low losses in many materials including the human body; (3) have (relatively) small wavelengths in water (λ ≈ 15 mm at 100 kHz, 1.5 mm at 1 MHz, and 0.15 mm at 10 MHz), making them useful for interactions with small systems; (4) have short time scales (τ ∼ 1/*f*), making them useful for exchanging energy with fast phenomena; and (5) can be produced by many well-established transducer technologies across the frequency range and with large ranges of excitation pressures. Because the acoustic displacements themselves are rather small at high frequencies, it is instead often the very high accelerations associated with ultrasound that can drive strong effects in microscale systems.^14^

Depending on the system that is being analyzed, it is convenient to convert between the different acoustic quantities described above. These properties can be related to each other explicitly depending on the wave geometry. In most cases of interest, the wavefronts are planar and propagate in one direction (like those illustrated in Figure 2). In this case the acoustic quantities are related by^12^
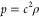
4
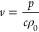
5
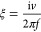
6where  is the imaginary unit. Equation 4 is derived from the equation of state of the material and indicates that the acoustic density fluctuations ρ are in phase with the acoustic pressure *p*: regions of high pressure correspond to compression in the medium and regions of low pressure correspond to rarefaction as described previously. For a plane traveling wave *p* and *v* are in phase as well, a fact that changes for curved wavefronts or close to interfaces. The particle motion ξ associated with the wave is proportional to the particle velocity, but 90° out of phase. Moreover, the particle displacement decreases as the acoustic frequency *f* increases for a fixed pressure. A *p* = 10 kPa plane wave in water causes particle motions of 1 nm at 1 MHz and 10 nm at 100 kHz, while a 1 MPa plane wave at those frequencies causes motions of 100 nm and 1 μm, respectively. By contrast, the density fluctuation and particle velocity are frequency independent. A 10 kPa pressure wave in water is associated with a particle velocity of *v* = 6.75 mm s^–1^ and a relative density change of ρ/ρ_0_ = 4 × 10^–6^. At 1 MPa, *v* = 675 mm s^–1^ and ρ/ρ_0_ = 4 × 10^–2^.

As was described above, acoustic waves carry energy, which can be used to perform useful work. The energy density carried by a plane wave is given by^12^
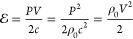
7where *P* and *V* are the acoustic pressure and velocity amplitudes.

Another commonly used metric for describing energy propagation in a wave is the wave intensity, which quantifies the rate of energy transfer by the acoustic wave (in units of W cm^–2^). The time average intensity for a plane wave in a fluid can be calculated directly from the pressure and fluid properties:
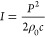
8

The strength of an acoustic field is sometimes reported as a pressure and sometimes as an intensity, depending on the application or conventions in a research field. Therefore, it is valuable to be able to convert between these two descriptions. Moreover, because energy must always be conserved, eq 8 is a useful tool to estimate pressure amplitudes in different systems, such as when sound is transmitted from a material with one set of material properties (ρ_0_, *c*) into another (ρ_0_′, *c*′). Similarly it helps to estimate the pressure produced by a transducer emitting a power *W* from an active area *A*. In this case, the intensity should scale as *I* ∼ *W*/*A*, which allows us to estimate the pressure generated in the propagation medium with (ρ_0_, *c*). Exemplary values for acoustic pressure and intensity in water are given in Table 1 as a reference, since most of the systems described in this review occur in an aqueous environment.

**Table 1:** Relationship between Acoustic Intensity and Pressure for Plane Waves in Water

pressure	intensity
1 kPa	3.3 × 10^–5^ W cm^–2^
10 kPa	3.3 × 10^–3^ W cm^–2^
100 kPa	0.33 W cm^–2^
1 MPa	33 W cm^–2^

After considering how acoustic waves carry energy through materials in the form of pressure, density, and local velocity fluctuations, we will discuss how different materials and geometries impact the acoustic response of a system and the associated energy transfer.

### 2.2 Controlling Acoustic Waves

#### 2.2.1 Acoustic Properties of Materials

The acoustical and geometrical properties of a medium dictate how effectively energy can be transported between two points. This also opens up opportunities to control the wave and energy propagation by structuring materials appropriately. Commonly, a material’s acoustic properties are described by its acoustic impedance *Z* = ρ_0_*c*, which describes the resistance of a medium to move given a fixed pressure excitation (see eq 5). A high-*Z* material will exhibit smaller particle velocity for a fixed excitation pressure. Conversely, a fixed vibration amplitude of a surface (such as a transducer) will produce lower pressures in a low-*Z* material. The acoustic impedance determines the behavior of waves at the interface between different media. If an acoustic wave is incident on a flat interface between two media with impedances *Z*_1_ and *Z*_2_, sound will reflect backward from the interface with a reflection coefficient:
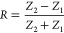


The fraction of the incident energy reflected back from the interface is given by |*R*|^2^. Acoustic waves couple more efficiently from one medium to another if their impedances are similar, so *R* → 0. The acoustic impedance therefore determines how strongly acoustic waves are scattered or reflected from an interface, and this can have important implications when designing acoustic systems such as resonators or when trying to control materials with sound, e.g., using the acoustic radiation force, as will be seen below. As a reference, the values of ρ_0_, *c*, and *Z* are provided for some common materials in Table 2. To improve acoustic transmission between highly disparate media, as often encountered in transducer design, matching layers can be used. For example, ideal transmission can be achieved between two interfaces by adding a thin layer between them, with impedance  and thickness λ_3_/4 (where λ_3_ = *c*_3_/*f*). The thickness constraint restricts this concept to work only in a narrow frequency band. In practice, two or more consecutive matching layers are often used to provide more robust broadband matching. Alternatively, broadband matching layers have been reported using gradient-index metamaterials, which are structures with subwavelength features that change the bulk properties for acoustic waves (cf. gradient index materials in optics).^15^

**Table 2:** Acoustic Properties of Selected Materials at Room Temperature (20 °C)^a^

material	ρ (kg m^–3^)	*c* (m s^–1^)	*Z* (Pa s m^–1^)	*a* (dB cm^–1^)	notes	ref
air	1.2	343	412	–	20 °C	(12)
water	997	1482	1.5 × 10^6^	2.2 × 10^–3^	at 100 kHz	(18, 19)
				0.22	at 1 MHz	(19)
brain tissue	1035	1562	1.6 × 10^6^	0.58	at 1 MHz, 37 °C	(20)
bone	1990	3198	6.4 × 10^6^	3.54	at 1 MHz, 37 °C	(20)
glycerol	1260	1904	2.4 × 10^6^	–		(21)
silicone oil	818	960	0.8 × 10^6^	–	Dow 200, 1 cSt viscosity	(21)
PDMS	1031	1028	1.1 × 10^6^	0.4	at 3 MHz	(22)
PMMA	1191	2690	3.2 × 10^6^	0.7	at 1 MHz	(23)
silica glass	2200	5900	13 × 10^6^	–		(24)
brass	8470	4494	38 × 10^6^	–	C38500 alloy	(24)

a ρ, density; *c*, sound speed; *Z*, impedance; *a*, attenuation coefficient.

An additional important material property when designing acoustic systems is the material attenuation coefficient. Attenuation describes the loss of acoustic energy irreversibly to heat through various mechanisms such as viscosity or molecular relaxation.^12^ When an acoustic wave propagates in material, the pressure amplitude after a distance *L* is given by *p* = *p*_0_e^–*αL*^, where *p*_0_ was the initial pressure of the wave and α is the attenuation coefficient in neper per centimeter. More commonly, the attenuation coefficient is expressed as *a* = 8.7α, which is given in units of decibels per centimeter. Attenuation depends strongly on frequency, and higher frequencies are attenuated more strongly than low frequencies. Empirical models typically describe this dependence as a power law: *a*(*f*) = *a*_0_*f*^γ^, where 0 < γ ≤ 2. To describe a material’s attenuation at all frequencies, it is therefore sufficient to know the value of *a* at one frequency, the frequency at which *a* was measured, and the power γ. For liquids including water γ = 2,^16^ while for many polymers including PMMA the relation is almost linear (γ ≈ 1).^17^Table 2 provides the attenuation coefficient *a* and measurement frequency for a few select materials.

#### 2.2.2 Effects of Geometry on Acoustic Waves

**Figure 3:**
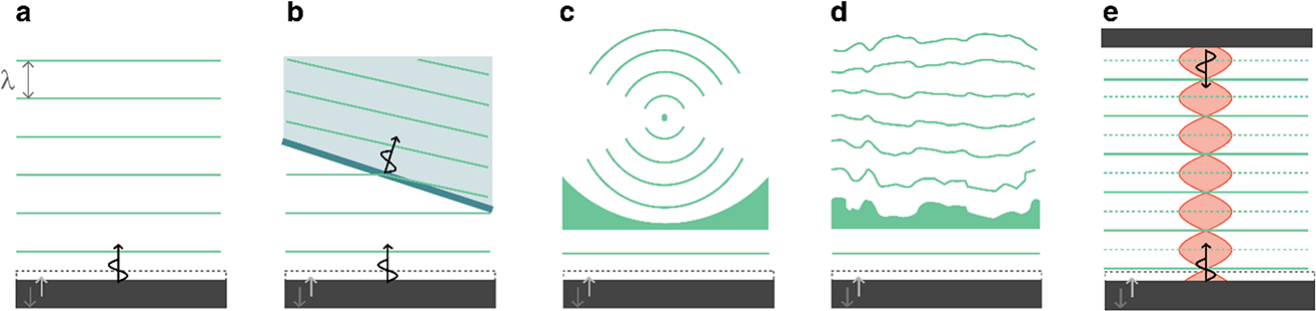
Methods to shape ultrasound fields. (a) A simple piston transducer approximately produces a plane wave in a uniform medium. (b) When the plane wave transmits into a medium of a different sound speed, refraction causes the wave direction and wavelength to change. (c) A lens can be used to focus plane waves to a point. (d) An acoustic hologram provides more control to turn a plane wave into an arbitrarily shaped pressure field. (e) A resonator can be built to create a high-amplitude patterned pressure field using two opposing transducers or a transducer and a reflector.

In addition to the choice of material, a material’s geometry can also be used to control the propagation of acoustic waves. Ultrasound transducers are typically designed to emit *plane waves*, as shown in Figure 3a. When a plane wave is incident on the interface between two different materials, part of the energy will be reflected and part will be transmitted, as described above. When the interface is planar, the transmitted portion of the wave will bend, or *refract*, as shown in Figure 3b. Refraction depends on the sound speeds of the two media and the angle of incidence: stronger refraction will take place between two media with very different sound speeds. A nonplanar interface will lead to more complex refraction and reflection and, therefore, can produce more complex acoustic fields. For example, a curved interface can be used to focus acoustic waves, as shown in Figure 3c. While lenses are commonly designed for use in transmission as depicted here, it is also possible to use a curved reflective surface to focus sound as well. Refraction is not inherently frequency dependent; however, when curved interfaces are used, the focusing properties will depend on the relative magnitude of the focal length and the wavelength, making lenses frequency dependent. For a fixed lens geometry, higher-frequency ultrasound can be focused to a smaller spot than lower-frequency ultrasound.

Recently, powerful techniques have been developed to shape acoustic fields using acoustic holograms, as shown in Figure 3d.^25^ Acoustic holograms use algorithmically designed interfaces to shape an incident acoustic plane wave so that it forms a desired (and arbitrary) field shape after a certain propagation distance. Acoustic holograms have been used to create pressure fields patterned with very high complexity, and they can be tailored to each specific application much more flexibly than a conventional focusing element such as a lens. Similar to lenses, acoustic holograms can be designed for use in transmission geometries (as depicted) or in reflection, and the resolution of the features that can be produced increases with the frequency.

Interface geometry also plays an important role when more complex wave types are used. Surface acoustic waves (SAW) are waves that propagate only at interfaces between two media and can be used to confine and guide energy along a specific path, which is finding increasing application in microscale devices.^14^ When at least one of the media is solid, then the interface geometry can also strongly determine what kinds of waves can be reflected. In some cases, the geometry can prevent certain kinds of elastic or surface waves from being generated, while in other cases it can be used to efficiently convert energy between different propagating wave types.^13^ While such mode conversion is sometimes a source of undesired losses, it is also a common technique used when designing transducers to excite specific kinds of waves.^26^ Increasingly, custom surface designs (metasurfaces) are being explored to produce specific and tunable reflection or refraction behaviors.^27^ However, these techniques are currently used in the audible (low-kilohertz) frequency ranges, and scaling the structures down for use at megahertz frequencies remains an open challenge.

Finally, acoustic fields can be shaped and amplified by confining them in space within a *resonator*. Resonators can be designed in different shapes and configurations, but the simplest resonator is a homogeneous medium between two rigid walls, separated by a distance *L* = *nλ*/2 = *nc*/(2*f*), for any integer *n*. Such a resonator geometry is shown in Figure 3e. Acoustic waves in a resonator propagate in both directions—either because of reflection off one wall or because of being driven by two opposing transducers. The opposite-traveling waves interfere and produce a standing wave pattern, which is fixed in space. For the best performance, it is critical that the walls are properly spaced at the resonant distance. When this condition is satisfied, constructive interference leads to a strongly amplified field in the resonator, in the form of a regular grid of high- and low-pressure regions that can be used for different applications, such as trapping and manipulation of small objects. Because the performance is dependent on the boundary spacing *L*, resonators are typically designed to operate at one frequency.

#### 2.2.3 Bubbles

Because of their geometry and mechanical properties, gas bubbles constitute a special class of acoustic material that is used heavily in emerging ultrasound technologies. While they are not typically used to shape acoustic fields, their response to acoustic fields can be tuned and measured for different applications. Bubbles are unique acoustic objects because they can produce large acoustic and vibrational responses to sound whose wavelength is much larger than the bubbles themselves. For the description here and the discussion in the following sections, we will only consider subwavelength spherical air bubbles in water.

The mechanical properties of gas bubbles are responsible for their strong response. Gas bubbles are highly compressible and behave like a spring when excited by ultrasound: they store energy in compression and expansion cycles and release it as kinetic energy within the surrounding fluid. Bubbles are therefore resonant objects, whose response can be amplified by driving them at their resonance frequency. Resonant excitation leads to large amplitude motion of the bubble walls and a stronger scattering response for sensing. A resonant bubble can reflect over 100 times more energy than expected on the basis of its size alone.^28^

The fundamental resonant frequency of a bubble is known as the Minnaert frequency *f*_M_, which is set by the bubble size, properties of the gas, and properties of the fluid:^28,29^
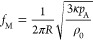
9

Here, *R* is the bubble radius, κ is the polytropic coefficient for the gas, *p*_A_ is the ambient pressure, and ρ_0_ is the density of the surrounding liquid. Additional factors, such as surface tension, shift the resonant frequency from this idealized value;^28,30^ however, the Minnaert frequency provides a good starting estimate for a bubble resonance in most cases. A 1-μm-diameter air bubble in water is resonant near 6.6 MHz; at 10 μm, the resonance drops to around 660 kHz, and at 100 μm it drops to 66 kHz.

The properties of the medium surrounding the bubble can also have a decisive effect on the bubble’s resonance. In many applications, small microbubbles are encapsulated in viscoelastic shells to stabilize them against gas diffusion. In other situations, bubbles may be embedded in a complex medium such as a gel. In these cases, the resonance frequency and resonant response will depend on the shell or medium properties as well.^31^ If a bubble is partially enclosed by a rigid structure, then it cannot expand symmetrically, and it will also exhibit altered resonance behaviors. When the bubble is enclosed in a cavity, higher-order interfacial resonances can be observed, where the bubble interface oscillates like a pinned membrane.^32,33^

Bubbles can be turned into tools by feeding them energy with an external sound field, causing the bubble to oscillate. These oscillations have two important effects. First, they can emit sound themselves (a scattered acoustic wave), creating a point source of sound that can be remotely measured. Second, they can generate strong fluid flows that can be used to apply fluid stresses to objects at the microscale and molecular scale. These streaming effects are often amplified in the presence of rigid boundaries and structures.

The strength of these different bubble responses depends primarily on the size of the bubble (which sets the resonance frequency) and the driving ultrasound frequency. For scattering, the strength is quantified by the scattering cross section of the bubble, which indicates how much of the incident energy is scattered by the bubble. At low frequencies (excitation *f* ≪ *f*_M_), the scattering cross section depends most strongly on the frequency, increasing as (*f*/*f*_M_)^4^. At resonance (*f* = *f*_M_), the cross section only depends on the resonance frequency of the bubble, scaling as *f*_M_^–2^ or, equivalently, as *R*^2^. The larger the bubble, the stronger its resonant response will generally be.^28^ The strength of streaming responses from bubble excitation is more complicated and also depends strongly on the presence of boundaries around the bubble. These effects are discussed more in sections 2.3.2 and 3.6.1. Another way to increase the response strength of a bubble is to increase the amplitude of a driving field. However, after a certain point, large vibration or pressure amplitudes will lead to nonlinear bubble oscillations^34^ and cavitation, which is discussed more in section 2.3.4.

Because they can convert acoustic energy into other forms of energy such as fluid flow, bubbles can also serve as sources of loss for acoustic waves. In general, bubbles will lose energy through one of three mechanisms: thermal losses during gas compression, viscous losses in the fluid, and acoustic scattering. Depending on the size of the bubble, different loss mechanisms dominate.^30^ For air bubbles in water that are larger than 10 μm, viscous forces are negligible, and thermal losses dominate at low frequencies while acoustic radiation dominates at high frequencies. For smaller bubbles, viscous losses become significant at low frequencies, but the high-frequency damping response is still dominated by acoustic radiation.^30^

### 2.3 Using Acoustic Energy

When ultrasound is used for smart systems, one important goal is to use the acoustic waves for nonacoustic work, such as moving objects, initiating chemical reactions, and mechanically triggering biological processes. In order to achieve this, it is necessary to convert the acoustic energy. Sometimes this is because the final action is inherently another form of work, e.g., electrical or chemical. Other times, it is because the target system cannot respond strongly to mechanical stimuli at ultrasonic frequencies. In either case, mechanisms are required that can convert the acoustic energy into a more useful form for the task at hand. In this section, we describe four mechanisms that are commonly used for this purpose: piezoelectricity (section 2.3.1), acoustic streaming (section 2.3.2), acoustic radiation forces (section 2.3.3), and cavitation (section 2.3.4).

#### 2.3.1 Piezoelectricity

Piezoelectric materials are noncentrosymmetric materials that generate an internal electrical polarization in response to an applied mechanical stress. Consequently, the (inverse) piezoelectric effect can be used to produce motion and therefore acoustic waves from an externally applied electrical voltage. Piezoelectricity can be observed in crystals such as quartz, ceramics such as lead zirconate titanate (PZT), and polymers such as polyvinylidene fluoride (PVDF). The strength of the piezoelectric effect can be quantified by the longitudinal piezoelectric coefficient *d*_33_, which describes how much the material will deform for a given voltage. A typical value for commercial PZT is *d*_33_ = 265 × 10^–12^ m V^–1^ (PIC181, PI Ceramic, Germany). In addition to being useful for converting electricity into motion, piezoelectric materials can also convert sound into electrical signals via the direct piezoelectric effect. Beyond applications for sensing ultrasound, the direct piezoelectric effect can be used to generate voltages that can be used to trigger chemical processes and biological signaling, as discussed in section 3.

#### 2.3.2 Acoustic Streaming

**Figure 4:**
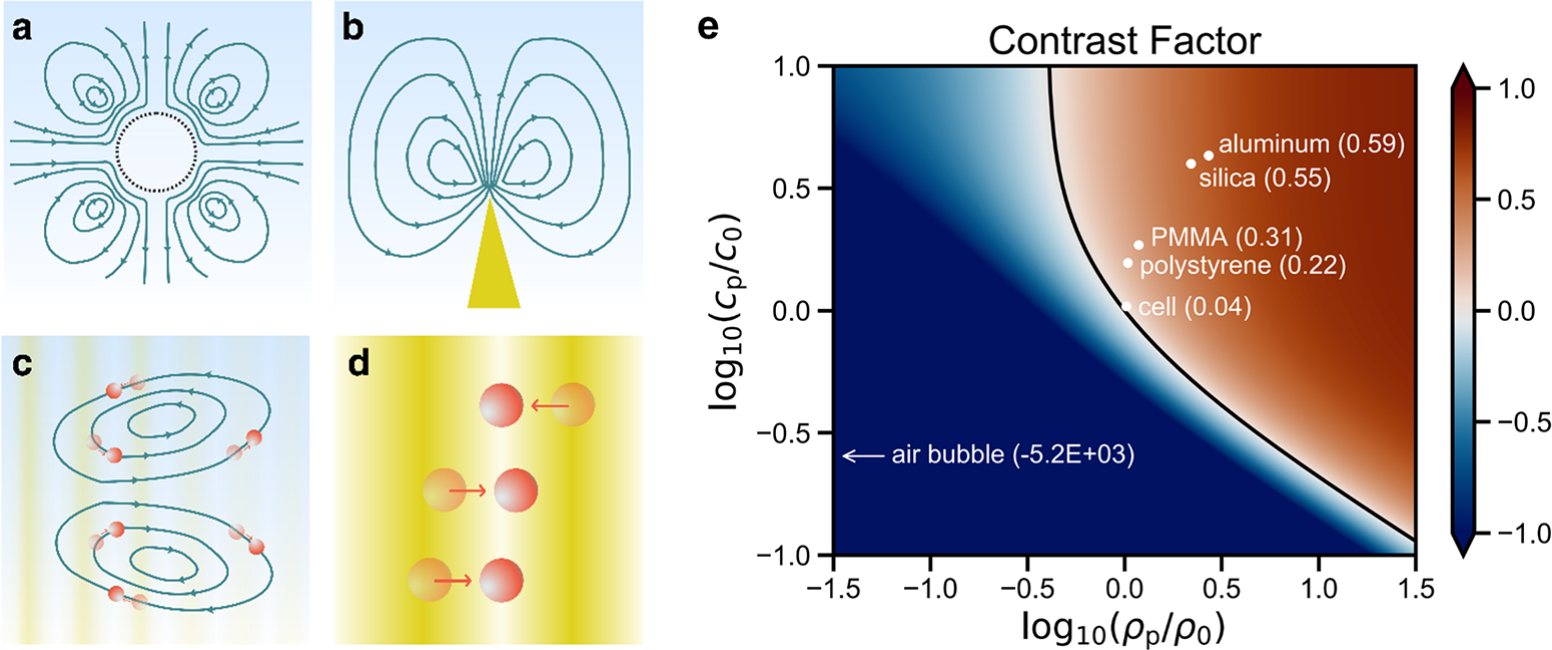
Different mechanisms can be used to convert ultrasonic energy into other forms for useful work. (a) Bubble vibration induced streaming. (b) Microstructure vibration induced streaming. (c) Acoustic streaming induced by acoustic wave propagation and attenuation. (d) Acoustic radiation force. (e) Acoustic contrast factor as a function of particle and fluid properties, with specific values plotted for common acoustic materials in water.

In order to directly convert high-frequency ultrasonic waves into steady forces on objects, it is necessary to make use of nonlinear acoustic mechanisms. The first nonlinear acoustic mechanism that can be used for this purpose is acoustic streaming.^35^ Acoustic streaming refers to fluid flow driven by acoustic waves, which is caused by momentum transfer from the acoustic waves to the fluid. This can occur at the fluid boundary layer along vibrating bubbles or structures, where the dissipation of acoustic energy due to the steep velocity gradient induces boundary layer streaming, called Schlichting streaming.^36^ Driven by the shear of this boundary layer streaming, there will be a flow in the bulk fluid called Rayleigh streaming, as shown in Figure 4a,b.^37^ Acoustic streaming can also occur in a bulk fluid because of attenuation of the propagating wave. This is known as Eckart streaming and is shown in Figure 4c.^38^

Rayleigh streaming is strongest when a structure, such as a bubble or a beam, is excited on resonance, causing maximal vibrations. The resonance frequencies of elastic structures are dependent on their size, geometry, and mechanical properties. When driven by a fixed acoustic intensity, high-aspect-ratio or sharp-edged structures generally provide a stronger streaming response than low-aspect-ratio structures. The oscillation of these resonant microstructures will induce intense Schlichting streaming in the surrounding fluid boundary layer, which will generate strong Rayleigh streaming in the nearby bulk fluid.^39^ Rayleigh streaming can also happen when a surface acoustic waves propagate along a solid boundary, which typically has a higher frequency than the resonant microstructures.^14^

In the presence of acoustic streaming, any structures or particles in the flow will experience a drag force caused by the viscosity in the fluid. At small length scales, the fluid flow is typically dominated by viscous effects, and the viscous stress applied to a boundary by a fluid moving with velocity *v* and viscosity μ is given by τ = μ∇*v⃗*. The shear stress from a fluid is proportional to viscosity and to the gradient of the velocity, which is also known as the strain rate. Small objects in the flow, such as microparticles, will be carried along with the flow unless they are held in place by other forces (e.g., magnetic, electrostatic, or acoustic radiation forces). Soft structures and macromolecules, on the other hand, can be deformed by the drag forces, or even broken by them if the shear rate is high enough.^40^

#### 2.3.3 Acoustic Radiation Forces

The second nonlinear mechanism that can be used to create steady forces is the acoustic radiation force (ARF).^41^ Acoustic radiation forces can be experienced by surfaces, structures, or microparticles exposed to ultrasonic waves (see Figure 4d). Most commonly, we will discuss the acoustic radiation force on microparticles that are much smaller than the acoustic wavelength.

For a uniform spherical particle suspended in a liquid, the ARF is dependent on the properties of both the acoustic field (i.e., the intensity and frequency) and particles (i.e., the size and acoustic properties relative to the surrounding media). Following a classic model for the acoustic radiation force,^42^ the ARF on an elastic spherical particle can be calculated as the gradient of a potential *F*_ARF_ = −∇*U*_ARF_, which is in turn proportional to the particle volume *V*, acoustic contrast factor Φ of the particle, and the acoustic intensity *I*: *U*_ARF_ ∝ *I*Φ*V*. The acoustic contrast factor measures the difference in acoustic properties between the particle and the surrounding fluid and is given by^43^
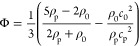
10where the subscripts “p” and 0 refer to the particle and the surrounding medium, respectively. The acoustic contrast factor is plotted in Figure 4e as a function of ρ_p_/ρ_0_ and *c*_p_/*c*_0_. The values of Φ for selected materials are labeled as well. As seen in the plot, the amplitude of the acoustic contrast factor—and therefore the strength of the ARF—increases as the particle and liquid become more acoustically different. For example, in the same acoustic field, the acoustic radiation force on a silica microparticle will be higher than that on a same-sized polystyrene microparticle. In comparison, a cell, which is mostly water, will experience a much lower ARF than either of the solid microparticles.

In general, materials with positive acoustic contrast (Φ > 0) move against a spatial pressure gradient and eventually accumulate in the pressure nodes (where *p* = 0), as shown in Figure 4d. Materials with negative acoustic contrast on the other hand are attracted to antinodes (where the pressure amplitude is maximal). Particle manipulation and directed assembly or tweezing at fixed locations can thus be achieved by carefully designing structured acoustic fields, e.g., by using a lens,^44^ hologram,^25^ diffractive element,^45^ or resonator.^46^

It should be noted that this model of the ARF only applies to simplified conditions where the particle diameter is much smaller than the acoustic wavelength of the medium and the shear acoustic waves in the particles are neglected. When these assumptions cannot be satisfied, more complex models can provide more accurate predictions for the ARF.^47^

A special case of ARF is experienced by bubbles in an acoustic field and can be broken into two parts: the primary and secondary Bjerknes forces.^28,48,49^ Primary Bjerknes forces arise on an isolated bubble in an acoustic field and are given by *F⃗*_B1_ = −*V*∇*P*, where *V* is the bubble volume, *P* is the acoustic pressure amplitude, and ∇ is the spatial gradient operator. Primary Bjerknes forces arise from slight differences in the pressure that a bubble experiences at different points in its oscillation. The primary Bjerknes force pushes bubbles toward regions of high pressure when they are excited below resonance (bubble smaller than the size resonant with driving field) and toward regions of low pressure when they are excited above resonance (bubble larger than resonant size). The secondary Bjerknes force emerges between two or more bubbles in an acoustic field, through the pressure fields scattered by each bubble. The secondary Bjerknes force can be attractive or repulsive depending on the bubble sizes and the driving frequency. Because the secondary Bjerknes force depends on the scattered field, it is shorter range and typically weaker than the primary Bjerknes force. However, when many bubbles are aggregated within a region of low pressure, such as at the nodes of a resonator, the secondary Bjerknes forces can play an important role, leading to motion, rearrangement, and clustering behavior of the bubbles. Elastic particles can experience similar secondary radiation forces based on scattered waves.^50^

#### 2.3.4 Cavitation

A final and important mechanism to convert acoustic energy into other forms is cavitation.^51^ During cavitation, an acoustic wave causes bubbles to form, oscillate, and potentially collapse in a fluid. Because of the strong response of bubbles to ultrasound, significant amounts of energy can be transferred from an ultrasound wave into bubble motion during the cavitation process. Between the large amplitude motion and the violent bubble collapse, cavitation can provide both strong mechanical and thermal stimuli. Additionally, during bubble collapse, a small plasma can form (sonoluminescence), which can also play an important role in optical and chemical processes. During bubble collapse, the temperature can briefly (<1 μs) reach several thousand kelvin and pressures on the order of tens of megapascals. These extreme conditions are associated with plasma formation in the collapsing bubble, which can produce radical species and emit electromagnetic radiation known as sonoluminescence.^52^

Cavitation occurs when the rarefaction (negative) pressure in an acoustic wave is low enough to draw dissolved gases out of solution into bubbles. This effect is more likely at lower frequencies, where the duration of strong negative pressure is longer each cycle than at higher frequencies. If nucleation sites are present in the fluid, such as micro- or nanoparticles, or rough surfaces, then cavitation can typically occur more easily. Similarly, if a fluid already contains microbubbles, then the effects of cavitation can be observed more easily. Conversely, cavitation effects can be suppressed by degassing the fluid.

A metric that indicates the strength of cavitation effects in aqueous systems with microbubbles is the mechanical index (MI):^28^
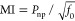
11where *P*_np_ is the peak negative pressure in an acoustic wave in MPa and *f*_0_ is the frequency in MHz. While the MI is calculated in units of , it is commonly reported without the units. At low MI (MI < 0.1), microbubbles only scatter the ultrasound signal (linear backscattering). At intermediate MI (0.1 < MI < 0.4) the bubble response becomes nonlinear, with increased scattering at harmonic and subharmonic frequencies^34^ (integer multiples or fractions of the driving frequency), due to large stable volumetric oscillations. This regime, which is referred to as stable cavitation, can induce slow bubble destruction via diffusion depending on the gas solubility in the surrounding medium. At higher MI (MI > 0.4), microbubbles will violently oscillate to the point of their collapse, emitting acoustic waves in a wide range of frequencies. This regime is known as inertial (or unstable) cavitation.

Different mechanical effects can be induced by ultrasound via cavitation, depending on the MI.^53^ The linear and nonlinear re-emission of sound at low to intermediate MI is the basis for the use as ultrasound contrast agents.^54^ Additionally, the increase in volumetric oscillation amplitude at intermediate MI is responsible for microstreaming flows.^55^ Bubble collapse at high MI may be accompanied by the emission of a shock wave.^52,55^ Microstreaming and collapse both will impose shear stresses on nearby structures. If the bubble is close to a surface, such as a container wall or another bubble, it can exhibit highly nonspherical oscillations that at intermediate MI values give rise to microstreaming velocities on the order of 1 mm s^–1^.^55^ At higher MI, collapsing bubbles near a surface can generate a liquid microjet that can cause damage to solid structures.

In addition to inducing mechanical effects, cavitation can also convert acoustic energy into heat.^55^ Three mechanisms may be involved in heat generation, depending on the size of the bubble, ultrasound parameters, and viscosity of the medium. The first one is heating of the surroundings due to the nonlinear acoustic radiation. The second effect is heating through viscous dissipation in the liquid during bubble motion. The third effect is thermal conduction through the gas core during compression of the bubbles. Heating due to nonlinear acoustic emission typically dominates in biomedical applications.^55^

#### 2.3.5 High- and Low-Intensity Ultrasound

The nonlinear effects described in this section all typically require high pressures or intensities to be realized. The use of high-intensity ultrasound often comes with additional instrumentation challenges and risks for damage, e.g., in sensitive biological systems. Therefore, it is often desirable to operate with lower power acoustic systems when possible. However, there are no clear boundaries between high- and low-intensity (or power) ultrasound, and different definitions are adopted by different authors. Here, we will avoid arbitrarily defining a boundary between low- and high-intensity ultrasound, and instead we will always provide the intensity or pressure levels in our descriptions.

Nonetheless, certain metrics are still valuable reference points for high-intensity ultrasound. As described above, the mechanical index can indicate when different detrimental effects of cavitation can be expected. In addition, guidance from the United States Food and Drug Administration (U.S. FDA) regarding safe operating levels for medical ultrasonic devices^56^ is often adopted as guidelines for other applications. The FDA defines maximum allowable intensities based on the MI and two intensity metrics: the spatial-peak pulse-average intensity (*I*_SPPA_) and the spatial-peak temporal-average intensity (*I*_SPTA_). *I*_SPPA_ is the time-average intensity over the duration of a pulse, whereas *I*_SPTA_ is the time-average over a longer time frame, therefore applying to continuous excitation as well. The maximum allowed intensities depend on the target tissue, but maximum permissible values for peripheral vessels are defined as *I*_SPTA_ = 720 mW cm^–2^ and *I*_SPPA_ = 190 W cm^–2^ or a mechanical index MI = 1.9. Higher values result in a temperature increase via absorption and thus can lead to cell death above 43 °C.

In section 3, acoustic effects and properties described here will be built upon to shape, trigger, interrogate, and actuate materials.

## 3 Acoustic Responses for Smart Materials

### 3.1 Patterning and Assembly of Biological Materials

Living biological matter can be seen as a blueprint for smart materials, as it can self-organize and act in response to cues from the environment. It is therefore of interest to build new hybrid smart materials from living components, such as cells. However, patterning and assembling functioning cellular structures—let alone synthetic organs—using biological building blocks is still a major challenge.^57,58^ Acoustic waves are benign to cells and can be used to assemble and shape biological matter via fluid streaming or the acoustic radiation force. The resulting forces can move biological cells and push them into predefined locations. It is also possible to align and assemble different cell types into functioning cell aggregates and simple organoid-like structures. These acoustic bioassemblies are promising for biomedical research, including drug screening,^59^ tissue engineering,^60,61^ and disease modeling.^62^

Acoustic assembly techniques complement other techniques that have also been developed for these purposes, and they generally offer advantages for assembly in certain circumstances. For instance, optical tweezers have found widespread use in the manipulation and assembly of individual cells and small particles. A recent review by Dholakia et al.^10^ highlights some of the key differences between optical and acoustic techniques for manipulation. Ultrasound is shown to generally provide higher trapping forces than light, while sacrificing some of the force sensitivity and spatial precision of optical techniques. Acoustic patterning and assembly also offers the advantages of good biocompatibility, rapid and parallel control of large numbers of cells, and efficient transmission for long-range control of assembled systems.

**Figure 5:**
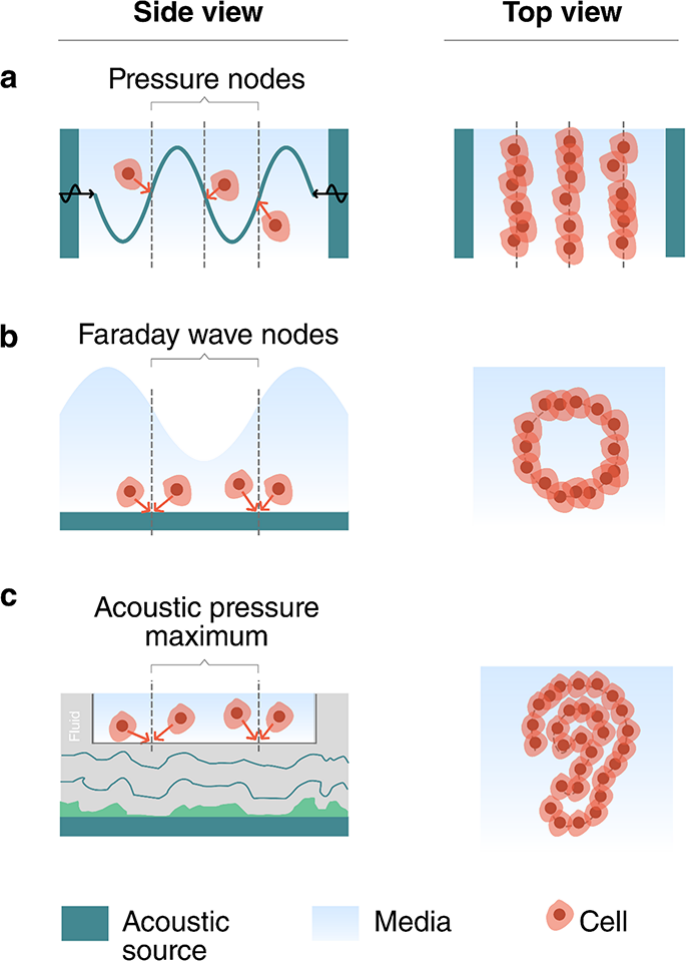
Acoustic patterning and bioassembly. (a) Standing wave trapping of cells in a resonator, (b) Faraday wave patterning, and (c) holographic acoustic patterning.

The acoustic methods that are predominantly used in the patterning and assembly of cells can be divided into three categories, as shown in Figure 5: standing wave trapping,^63,64^ Faraday wave patterning,^65−67^ and holographic patterning.^68,69^

Standing wave trapping (Figure 5a) is based on the acoustic radiation force experienced by cells when they are exposed to a standing acoustic wave. As described in section 2.2.2, standing acoustic waves are most commonly formed by opposing pairs of acoustic sources^63^ or by a resonant cavity that is excited by a single acoustic source.^70^ The acoustic radiation forces in these systems range from ∼1 pN to ∼10 nN. Biological cells will typically be trapped at the pressure nodes and, accordingly, form highly symmetric assemblies and periodic patterns.

Faraday waves (Figure 5b) are forced surface ripples that form at the liquid–air interface of a bounded liquid. They are typically generated by low-frequency vibrations (40–200 Hz) and display a vertical surface deformation, which causes recirculating flows in the fluid. These flows can carry suspended cells via the Stokes drag toward the stagnation points, which are located below the nodes of the surface waves. Faraday waves are enhanced when the excited at resonance frequencies of the container. Cells have been assembled into simple shapes, such as periodic straight or curved lines.^65^

Holographic particle patterning^9^ (Figure 5c) uses holograms to shape the acoustic field for the assembly of cells.^68^ Acoustic fields can be holographically patterned using a 3D-printed holographic phase mask,^25,71^ a phased array transducer,^72−74^ or a spatial acoustic modulator.^75^ Unlike standing waves and Faraday waves, holographic sources can potentially shape arbitrary complex fields that are independent of the container geometry. Thus, holograms can assemble cells into nonsymmetrical and irregular patterns. Both acoustic streaming and acoustic radiation forces can be used to aggregate cells in areas of high acoustic pressure.^68^

**Figure 6:**
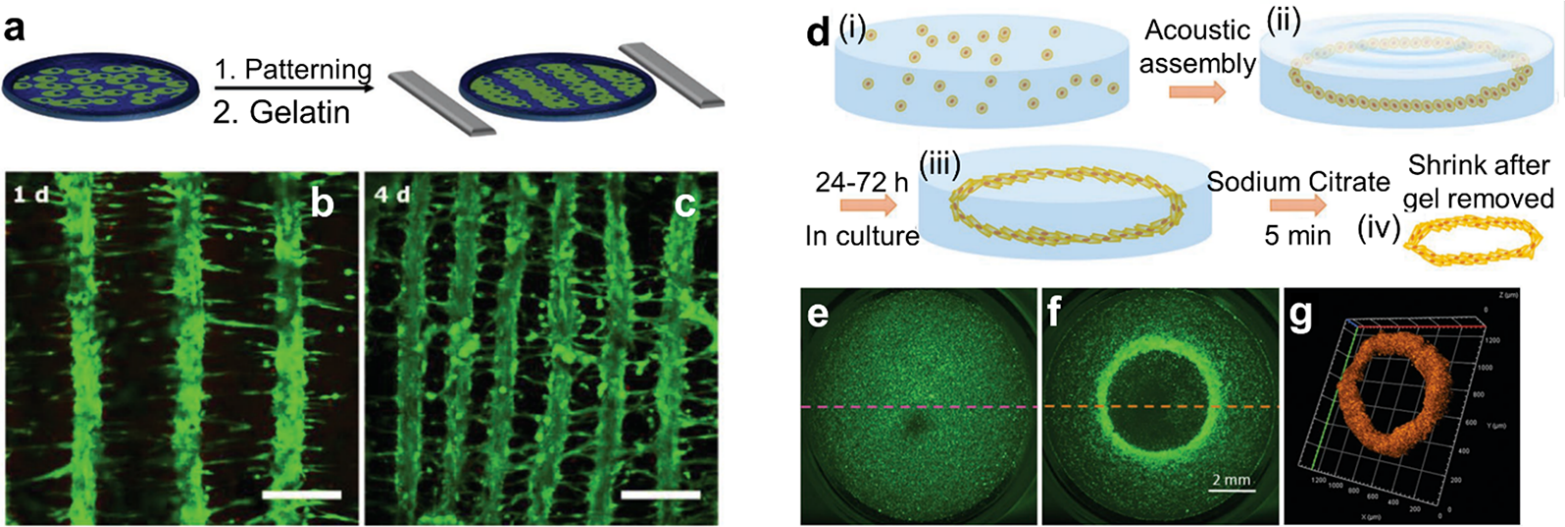
Acoustic assembly of cells to form actuators. (a–c) Patterning of fibroblast cells into stripes by standing acoustic wave trapping. Adapted from ref (60). CC BY 4.0. (d–g) Ring-shaped cell structures via Faraday wave patterning. Adapted with permission from ref (66). Copyright 2019 Wiley-VCH.

Patterning and assembling cellular structures offers the opportunity to use them as actuators and components in the development of miniaturized robotic systems.^76^ There has been considerable progress in using acoustic fields to align muscle cells (e.g., cardiomyocytes, myoblast cells) into tissuelike structures, which naturally actuate. Natural muscle tissue is organized along fibers, so the objective of the acoustic cell assembly is to form lines of cells. Armstrong et al.^60^ demonstrated acoustic assembly of myoblasts using standing acoustic waves (Figure 6a–c). Prior to the experiment the cells were suspended in a pre-cross-linked hydrogel. Then a standing pressure wave was generated between two pairs of opposing transducers to pattern the cells into periodic stripes whose pitch could be tuned by varying the frequency of the acoustic field. To immobilize the cell pattern, after assembly the hydrogel was cross-linked with UV light or slight heating. The cell patterns could then be incubated and formed tissue constructs with oriented multinucleated myotubes bundled into parallel, aligned muscle fibers. Tensile tests confirmed an increased Young’s modulus in the fiber direction. The assembled muscle tissue also responded to pulsed electrical stimulation. Ren et al.^66^ demonstrated the assembly of fibroblasts into ring-shaped structures using Faraday wave patterning (Figure 6d–g). The cell patterning was performed in a pre-cross-linked hydrogel (alginate), which was cross-linked after assembly with an ionic trigger (addition of calcium chloride) to immobilize the cell assembly for culturing. After 24–72 h the cells located on the outside of the ring showed radial alignment, while those on the inside showed circumferential alignment. The surrounding hydrogel could be dissolved and the cellular rings could be further assembled into tubular or concentric ring structures by using Faraday waves. The cellular assembly depends on the pattern of standing Faraday waves; thus its shape and size can be tuned by changing the container size or vibration frequency.^65^ This flexibility in cellular assembly could be potentially used in tuning the cell alignment and the mechanical properties of the assembly.

**Figure 7:**
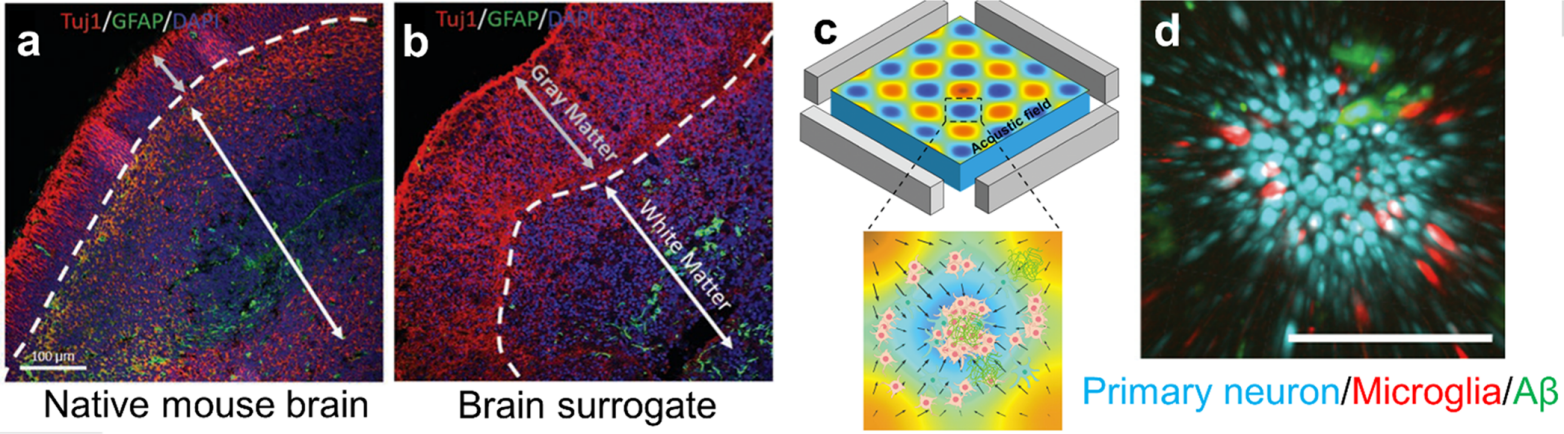
Acoustic assembly of neuronal and simple brain models. The bioassemblies show similar characteristics as the real organs. Native mouse brain (a) with an outer layer of neuron-rich cells and a neurite tract and a layer rich in glial cells. The acoustically assembled cells (b) exhibit similar features. Panels a and b reproduced with permission from ref (66). Copyright 2019 Wiley-VCH. (c, d) An acoustically aggregated brain organoid for Alzheimer’s disease modeling. Neuron inflammation depends on the presence or absence of amyloid-β in the environment. Reproduced with permission from ref (78). Copyright 2020 RSC Publishing.

Acoustic cell assembly has also been used for the development of simple cellular models for neural and brain studies.^77^ Organoids assembled from preselected cell types have been used to mimic specific brain regions, such as forebrain, midbrain, and cerebral cortex. The aforementioned work of Faraday wave cell patterning^66^ also demonstrated the acoustic assembly of simple brain organoids (Figure 7a,b). To mimic native brain tissue, the authors separately assembled neuron-rich and astrocyte-rich cellular rings, both from E18 mouse cells. Those were placed concentrically in a medium and cultured together for 14 days. The brain surrogate presented a viability of 87% after 7 days. The neurons in this simple brain organoid model showed synchronized calcium activity, indicating the formation of a network of neurons.

Acoustic bioassembly can be used to build disease models of brain tissue. Cai et al.^78^ used standing wave trapping to aggregate brain cells (including neurons, microglia, and astrocytes) and amyloid-β aggregates (potential key contributors to Alzheimer’s disease^79^) into spheroids to mimic the neuroinflammation process in Alzheimer’s disease (Figure 7c,d). Compared to the control group of spheroids without amyloid-β aggregates in the environment, the disease model spheroid showed a significantly higher expression of microglia activation, which is consistent with signs of neuroinflammation.^80^ Acoustically assembled spheroids can therefore potentially be used as convenient *in vitro* models for research into Alzheimer’s and other diseases.

The patterning and assembly of cells and living tissues using acoustics offers a versatile and benign route to construct biological smart materials, such as bioactuators and (brain) organoids. There is room for improvement to increase the complexity and functionality of the bioassemblies that can be generated with acoustic fields. Also, it is known that a 3D spatial control of the distribution of cells will extend the functionality^81^ and facilitate the integration of the bioassemblies with artificial microstructures.^82^ However, 3D control has yet to be shown with acoustic methods. In addition, some studies have shown that cells can assemble into spheroids in acoustic streaming flows.^83−87^ The flow enriches the cell concentration at the vortex center and also disturbs the cell adherence to the container surface; thus it enhances the formation of cellular spheroids. For future studies, precise and high-throughput control of the acoustic streaming profile could open up new directions in the patterning and assembly of biological materials. Going further, more studies can be expected to investigate the influence of acoustic waves on cellular properties that are important for long-term cell patterning and assembly. Such properties include proliferation,^88,89^ viability,^90^ metabolic activity, and differentiation. Finally, the ability to select the cell type during the assembly would open up the possibility to enable vascularization in the bioassembly,^91−93^ which is important for larger scale structures, and to realize the growth of real organs or tissues with acoustic fields.

### 3.2 Reconfiguring Shape and Material Properties

**Figure 8:**
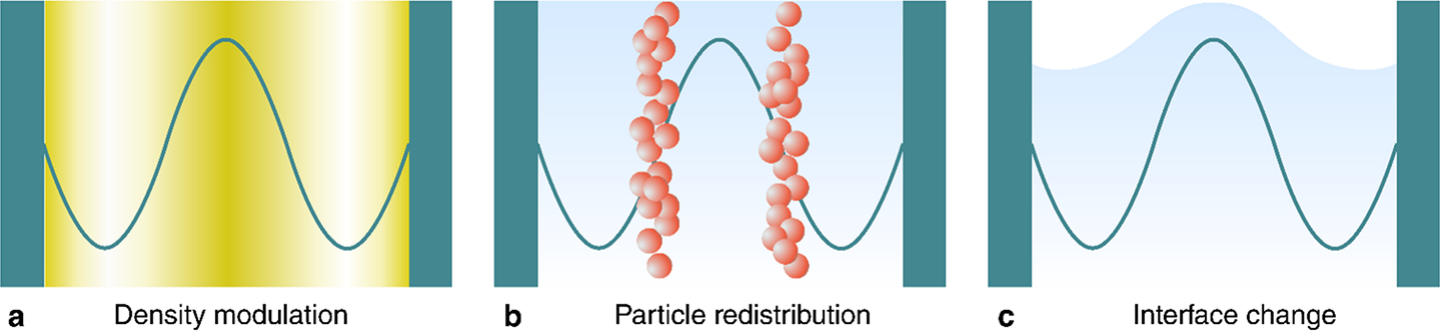
Three physical mechanisms provide control over different material properties: (1) pressure waves can modulate the density of a medium, (2) the acoustic radiation force on particles and molecules can modify the microstructure of materials, and (3) the acoustic radiation force can change the shape of an interface.

Acoustic fields can be used to change the shape and microstructure of a material, opening up pathways to control its functionality. Three physical mechanisms (Figure 8) provide a means to affect different material properties: (1) pressure waves can be used to modulate the density of a medium, (2) the acoustic radiation force on particles and molecules can be used to modify the structure and behavior of microstructured materials, and (3) the acoustic radiation force can change the geometry of interfaces. In this section we review how these mechanisms have been exploited to achieve different functionalities relevant to smart materials, focusing on examples where ultrasound has been used to control the propagation of light (section 3.2.1), the propagation of sound (section 3.2.2), and the mechanical properties of soft materials (section 3.2.3).

#### 3.2.1 Controlling Light with Sound

Smart systems can use ultrasound to control the propagation of light, e.g., for communication, computation, sensing, or power delivery. One of the biggest advantages of ultrasonic modulation in optical systems is the fast response time. For example, variable focusing techniques that rely on electrical, magnetic, or fluidic effects typically respond slower than 1 ms, whereas acoustic lenses and modulators can respond on submicrosecond time scales.^94−98^ Ultrasonic devices for controlling light ultimately rely on one of three fundamental mechanisms: causing spatial variations in a medium’s density, changing the shape of an optical interface, or patterning particles or orienting liquid crystals in a medium. Therefore, acousto-optic systems provide two key benefits for the development of smart materials. First, they reveal what kinds of structural and geometric changes can be controlled by ultrasound, and with what speed. Second, since the mechanisms take place at the microscale and even molecular scale, the techniques developed in optics provide insight into how smart systems could be designed to inherently manipulate light when exposed to ultrasound.

The most common acousto-optic systems rely on the photoelastic effect, whereby density changes from a pressure wave cause changes to the optical index of refraction. When light is transmitted through a region containing spatial variations in the index of refraction, the light will diffract and change its direction according to the pattern of the refractive index.^99^ By controlling the refractive index variations using ultrasound, light can be modulated, patterned, focused, or redirected.

The change in the index of refraction Δ*n* is related to the acoustic wave’s intensity *I*_ac_ by^100^
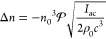
12where *n*_0_ is the medium’s refractive index without ultrasound applied,  is the medium’s photoelastic constant, ρ_0_ is density, and *c* is its sound speed. For example, for water *n*_0_ = 1.33,  = 0.31, ρ = 10^3^ kg m^–3^, and *c* = 1.5 × 10^3^ m s^–1^. Acoustic waves with an intensity of *I* = 10 W cm^–2^ will cause the optical refractive index to change by around 0.01%,^100^ which is small in absolute terms but large enough to achieve light modulation. The photoelastic response of water (e.g., as indicated by the  value) is strong compared to many optical materials; however, solid crystals have often been preferred because they show lower acoustic losses.^101^ Depending on the specific application requirements, different materials such as water-soluble oxides can provide both a strong photoacoustic response and the benefits of crystalline structure.^101^

The critical factors in controlling light with sound are the material properties of the propagation medium (especially the photoelastic constant and refractive index), the ultrasound intensity and frequency, and the geometry of the light-controlling system. Whereas the material properties and ultrasound intensity will affect the strength of the photoelastic response according to eq 12, the system geometry and ultrasound frequency will primarily determine how the light is scattered or redirected in any given application.

**Figure 9:**
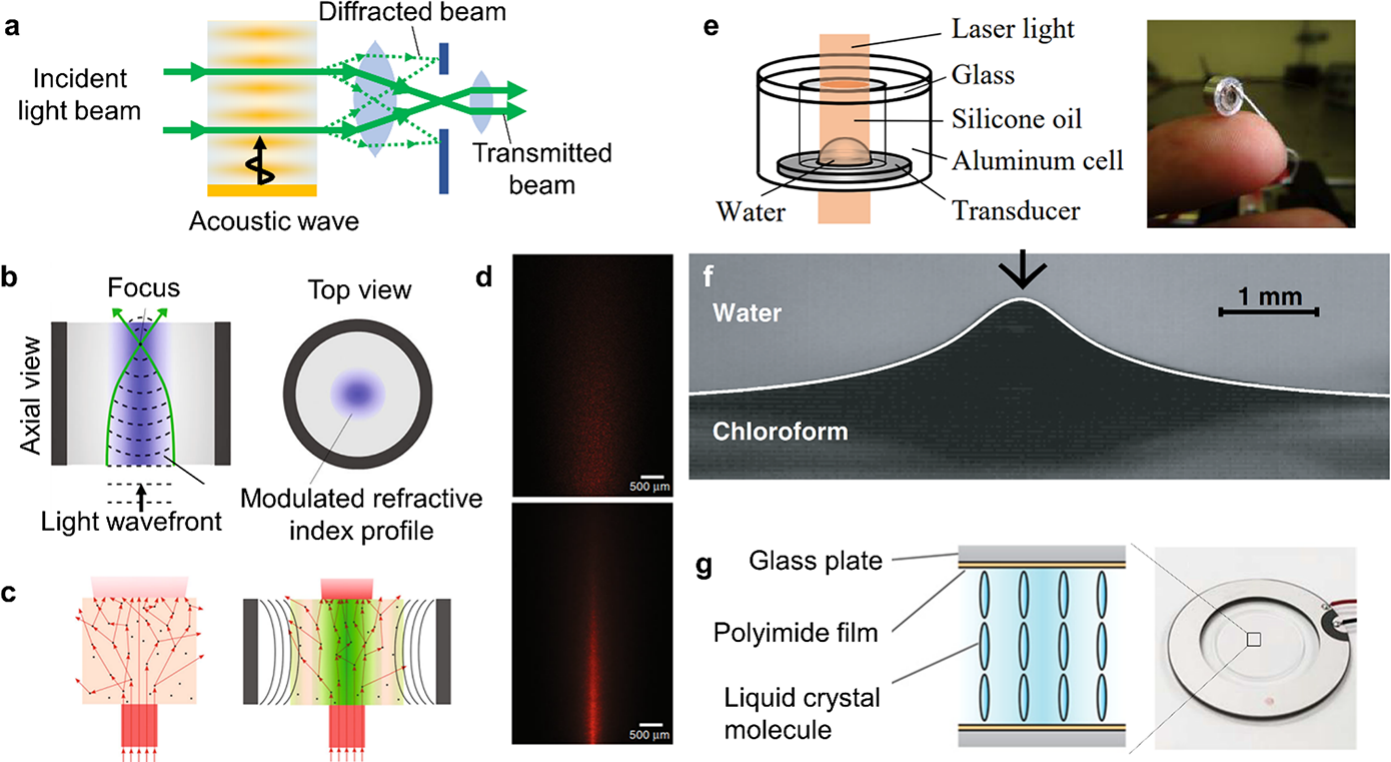
Working principles and examples of acoustically reconfigurable optical systems. (a) Acousto-optic modulator (AOM) used to control the propagation of light via ultrasound-controlled diffraction. The device can be used to redirect light or, with additional optics as shown here, to modulate the beam intensity. (b) Working principle of a cylindrical transient acoustic grating (TAG) lens. When the acoustic field (purple) causes density changes inside the resonator, the associated index of refraction changes bring incident light to a focus. (c) The principles of a TAG lens also help to focus light through a scattering medium. Optical scattering path without and with acoustic field applied. (d) Experimental images of light propagating through a TAG filled with scatterers without (above) and with (bottom) the acoustic field applied. Panels b–d adapted from ref (120). CC BY 4.0. (e) Two liquid deforming interface lens. Reprinted with permission from ref (129). Copyright 2010 OSA Publishing. (f) Large interface deformation activated by acoustic radiation force. Reproduced with permission from ref (130). Copyright 2008 EPL Association. (g) Liquid crystal lens operating principle and physical implementation. Adapted with permission from ref (131). Copyright 2018 AIP Publishing.

Using the photoelastic effect, different spatial patterns of sound can provide different kinds of control over light. Traditional acousto-optic modulators (AOMs) excite one-dimensional sinusoidal pressure waves in an optical medium.^102,103^ These variations create an optical grating that can diffract light, as shown in Figure 9a. The light can be scattered in different directions, as the grating period depends on the ultrasound frequency and amplitude. The switching rate between different states is limited only by the transit time of the sound wave through the AOM, leading to very fast modulation times, with rise times on the order of 10 ns possible.^104^ Depending on the optical system surrounding an AOM, the beam deflection can be used to create an optical switch,^103^ an optical power modulator,^105^ a phase modulator,^96^ a signal analyzer,^106^ a lens,^105,107^ or a beam deflector.^108^ The 1D geometry of the waves in traditional AOMs also makes it possible for ultrasound to filter the wavelength of light transmitted through the device, because of a phenomenon known as Bragg scattering.^109^ This capability has been used for applications in spectroscopy^103^ as well as communications, displays, and sensing.^110,111^ Additionally, the AOM can impart a small frequency shift to the incident light, which can be used for information encoding in sensing or communication applications.^110,112,113^ Since AOMs have been the subject of many comprehensive reviews, we direct the interested reader to standard references (refs (100−102, 105, 106, 110, 111, 114, and 115)) for more details on the design, operation, and applications of AOMs.

The photoelastic effect can also be used in a cylindrical geometry to create an adjustable lens. When a cylindrical resonator is excited with ultrasound at the frequency of a radial resonance, the standing wave in the resonator will vary in the radial direction but not along the cylinder’s length, as shown in Figure 9b. The optical index variations that are induced in this geometry give rise to a gradient index lens, whose focusing behavior depends on the amplitude and frequency of the ultrasound. Because they can be externally controlled by ultrasound, such devices are known as tunable acoustic gradient index (TAG) lenses. TAG lenses can be used to generate nondiffractive Bessel beams^116^ and to provide a variable focus lens for imaging.^117−122^ TAG lenses are typically constructed as water- or oil-filled cylindrical resonators. When the resonator is driven continuously with ultrasound, the focusing behavior of the lens will oscillate at the ultrasound frequency, and different focal lengths can be selected by synchronizing the light source or a sensor with the acoustic waves.^98,123^ Such systems have been used to image objects separated by 100 mm, with submicrosecond switching speeds,^97^ as well as for megahertz-frequency depth scanning in optical coherence tomography.^98^ Driving a 38-mm-diameter resonator at resonance (832 kHz), Scopelliti and Chamanzar^120^ demonstrated that the focal distance could be scanned over a range of 5.4 mm and the numerical aperture could be tuned by up to 21.5%.

A powerful benefit of using photoelastic methods to control light is that they can be applied directly to media of interest for sensing or power transmission through optically scattering media. For example, it has been demonstrated when ultrasound was applied directly to a tissue phantom in a TAG geometry. As shown in Figure 9c,d, the TAG focusing counteracted the scattering losses in the tissue and made an embedded object visible.^120^ For more flexibility, the resonator can be driven using an eight-element transducer array, which can switch the TAG lens between different focusing modes,^122^ enabling adaptive light delivery through real tissue.^121^ Another photoelastic technique that has been used for focusing light into scattering media is known as “acoustic guide star” focusing. When light passes through a region of focused ultrasound in a scattering medium such as tissue, it becomes phase shifted by the density variations and frequency shifted by the vibrating motion of the scatterers.^124^ This “tagged” light creates a virtual “guide star” whose emission can be measured and reversed to compensate for the scattering effects.^125^ Using collapsing microbubbles as the optical scatterers in acoustic guide star focusing makes it possible to focus light to below 2 μm through a tissue sample.^126^ Such light focusing and tagging techniques have been explored for imaging^125−127^ and neuromodulation,^128^ and could find additional uses in sensing, power delivery, or communication through highly scattering media.

The second class of acoustically reconfigurable optical components is based on the deformation of optical interfaces, such as water/oil,^129^ water/air,^132^ or water/gel^133,134^ interfaces. Since light refracts at the interface of two different materials, by controlling the shape of the interface it is possible to control how light is focused. Two approaches have been explored to generate such deformations with sound. One class of device uses the acoustic radiation force generated in a resonant cylindrical geometry filled with two liquids, as shown in Figure 9e. When the acoustic cell is excited, the radiation force deforms the interface between the two media, creating a lens whose shape and thus focusing power depend on the strength of the radiation force. By tuning the driving amplitude, the lens power can be shifted on the fly, with response times in the low-microsecond range.^129,133^ High-intensity ultrasound can also deform fluid interfaces in other geometries,^130,135^ as shown in Figure 9f. However, such extreme deformations are more difficult to use for optical control. An alternative approach to interface deformation is to use hydrodynamic motion associated with higher-order resonances in a cylindrical resonator. In these tunable lenses, the piezo drives hydrodynamic flows within the liquid, which lead to static interface deformations that can be controlled by the piezo driving voltage.^132^

The final class of acoustically driven optics relies on structural rearrangements of particles or molecules within an acoustic field. As described in section 2.3.3, acoustic fields can impart static forces on particles within the field through the acoustic radiation force. When particles are introduced into resonator geometries, such as the cylindrical TAG lenses, they will assemble and contribute to the optical focusing effects depending on their refractive index. Early reports using TAG lenses suggested enhanced focusing performance with nanoparticles in the resonator.^117^ Another way to make use of the acoustic radiation force is to place liquid crystals in a resonator. In the system described by Shimizu et al.,^131^ liquid crystals were trapped between two glass plates inside an ultrasonic resonator and were normally oriented perpendicular to the glass through chemical interactions, as shown in Figure 9g. When ultrasound was applied, the radiation force caused the liquid crystals to twist, changing the optical index of refraction. Since the ARF was strongest in the center of the resonator, the liquid crystal alignment varied as a function of radius, creating a lens whose focal length could be tuned by the ultrasound pressure (driving voltage). Applying modest powers up to 6.5 mW, the authors reported a shift in focal length of up to 1.2 mm from the 50-μm-thick liquid crystal layer. Liquid crystals dispersed in polymer droplets have also been used as ultrasound-switched shutters in a chip-based system excited by surface acoustic waves around 20 MHz.^136^ As the ultrasonic wave passes through the suspension, the liquid crystals reorient and no longer scatter light, creating a transparent window. However, with a response time on the order of 10 s, this approach is much slower than other acousto-optic techniques.

#### 3.2.2 Controlling Sound Propagation with Sound

The ability to control sound with sound is typically the domain of nonlinear acoustics, whereby high-intensity acoustic pressures alter the acoustic properties of materials enough to have an effect on the propagation of sound. Such effects can provide the ability to redirect sound^137^ or to generate new frequencies.^138^ However, nonlinear effects of sound in homogeneous media are relatively weak, because dispersion is basically absent and shock-formation processes dominate at ultrasonic frequencies of interest (<1 GHz). Reviews by Hamilton^138^ and Bunkin et al.^139^ discuss some nonlinear acoustic phenomena, often restricted to very high-intensity effects such as cavitation. Alternative approaches to achieve a strong nonlinear acoustic response, and thereby control sound with sound, have been developed based on structural effects. Examples have been demonstrated in waveguides and using combinations of highly dissimilar materials, such as ordered particle suspensions, gas bubbles in liquids, or cavities in solids.

**Figure 10:**
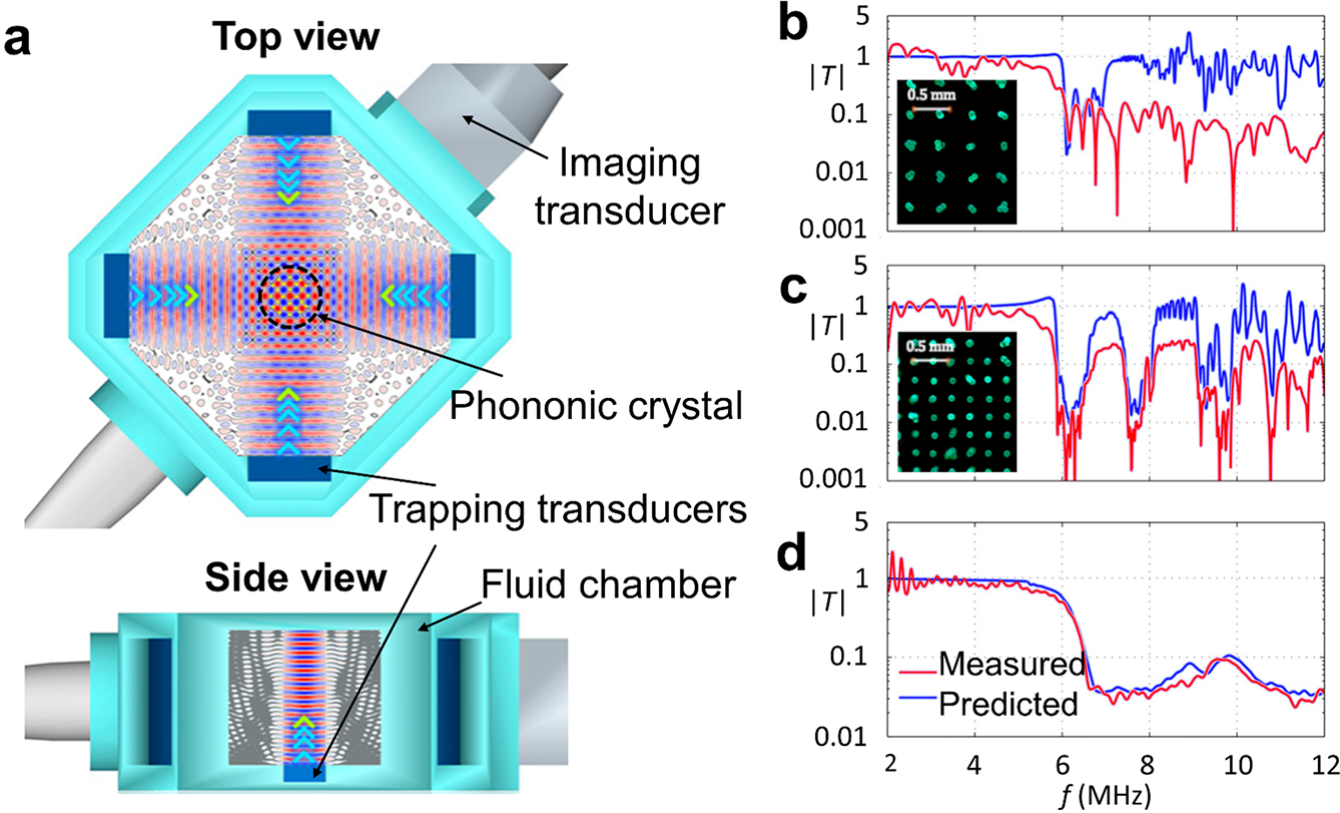
Reconfigurable phononic crystal formed by the acoustic assembly of polystyrene particles in water. (a) Top and side views of the experimental setup (rendering). Transmission plots through a phononic crystal formed (b) at 2.25 MHz, (c) at 3.75 MHz, or (d) for a random particle mixture. The insets in (b) and (c) show photos of the arranged particles. Adapted with permission from ref (140). Copyright 2014 the Authors.

A prominent example of such approaches is phononic crystals: materials that influence passing sound waves due to periodic features in their structures. Resonant inclusions in a material can block the propagation of sound within a specific frequency range, because all the energy is effectively trapped in the inclusion, leading to so-called band gaps. Scattering of a wave from periodically spaced features can similarly lead to constructive or destructive interference that depends on the feature spacing and the orientation. Phononic crystals guide or shape sound waves in unusual ways, and in this section it is seen how ultrasound can be used to modulate such behavior dynamically. For instance, Caleap and Drinkwater^140^ demonstrated how a reconfigurable phononic crystal can be obtained by assembling microparticles into a regular grid with the acoustic radiation force. Figure 10a shows a schematic of the setup, where the combined sound fields of three perpendicular standing waves formed a periodic grid of trapping sites in the center. The trapped particles assembled into a tetragonal crystal, where the lattice spacing could be tuned via the wavelength of the trapping field, in this case  and *a*_*z*_ = λ_*z*_/2. The properties of the phononic crystal can thus be changed in a dynamic and reconfigurable way. In this example, band gaps appeared in the acoustic transmission spectrum of a broadband ultrasound pulse traversing the crystal. The authors realized crystals at 2.25, 3.75, and 5.25 MHz of which 2.25 and 3.75 MHz are shown in panels b and c, respectively, of Figure 10 in comparison to the transmission through a random particle mixture (Figure 10d). It is evident that some parts of the spectrum show transmission while other ultrasound frequencies are blocked. In general, it should be possible to realize phononic crystals with lattice spacings ranging from 7.4 mm down to 74 μm in the 0.1–10 MHz range of ultrasound frequencies. More complex behavior can be expected for other crystal geometries, but the use of standing waves will restrict the number of accessible trapping geometries. Guevara Vasquez and Mauck^141^ investigated this problem theoretically for *D* spatial dimensions with the limitation that *N* = *D* pairs of opposing transducers are used.^141^ For *D* = 2 they found that three of the six possible Bravais lattice classes can be obtained by superposition of two standing waves, and for *D* = 3 they found 6 out of 14 possible classes. In two dimensions the available crystal geometries are orthorhombic centered, hexagonal, and tetragonal. In three dimensions the triclinic primitive, orthorhombic face-centered, trigonal primitive, cubic primitive, cubic face-centered, and cubic body-centered can be generated. More complex arrangements are to be expected if the number of transducer pairs exceeds the available dimensions *N* > *D*, but this has not yet been shown to date.

The acoustic radiation force has also been used to deform a water interface to make contact with a surface across an air gap, creating an acoustic diode.^142^ In addition to providing the ability to control sound, the performance of this system demonstrates how more extreme deformations can be achieved with ultrasound. Using pressures up to several hundred kilopascals, Devaux et al.^142^ showed that the water interface could be raised by at least 7 mm, with the interface height scaling as *h* ∼ ⟨*p*^2^⟩_*t*_ ∼ *A*^2^, where ⟨*p*^2^⟩_*t*_ is the time average of the input pressure squared, and *A* is the transducer excitation voltage. The response time of this device was in the range of hundreds of milliseconds and required gravity as a restoring force against the ARF, somewhat limiting its direct implementation in versatile smart systems.

#### 3.2.3 Controlling Mechanical Properties of Materials with Sound

An important ability of many biological materials that is sought after in emerging smart materials is their ability to switch between rigid and soft states quickly. To this end, recent work by Gibaud et al.^143^ demonstrates how ultrasound can be used to tune the mechanical behaviors of soft materials. Using soft colloidal gels (elastic moduli *G*′ = 0.1–10 kPa), Gibaud et al.^143^ showed that ultrasound exposure led to a rapid and significant, yet reversible, reduction of the gel’s elastic modulus. Depending on the gel, the modulus dropped by up to 80% when exposed to ultrasound between 20 and 500 kHz with a pressure of 150 kPa (intensity *I* = 1.5 W cm^–2^). Although the gels softened in the presence of ultrasound, they remained solid and did not fluidize in bulk. However, ultrasound exposure reduced the yield stress of the gels and accelerated shear-driven fluidization. After the ultrasound was turned off, the gel properties slowly relaxed to their original equilibrium stiffness, indicating that the effects are reversible. The softening and stiffening processes occur on the order of tens of seconds. The softening effects in the gels were shown to be strongly dependent on ultrasound power, but not on the driving frequency within the range tested. On the basis of these results and additional X-ray scattering measurements, Gibaud et al.^143^ proposed that the softening process is driven by thermally assisted microcrack formation in the gel network. While the field of ultrasonically controlled mechanical materials is in its infancy, these initial results indicate the potential for controlling the mechanical response of soft systems using ultrasound.

### 3.3 Sensing

Interaction with the environment is a key feature of smart materials,^144^ where sensing capabilities play an important role. Sensing can provide stimuli for direct action and feedback in adaptive systems. This section highlights ultrasound responses that probe a system’s mechanical (section 3.3.1), electrical (section 3.3.2), or biological properties (section 3.3.3).

#### 3.3.1 Bubble-Based Sensing of Mechanical Properties

The behavior of bubbles in a liquid or soft elastic body is directly linked to the mechanical properties of the medium (see section 2.2.3).^11,145^ Two different bubble responses can be used as measurement techniques: bubble translation from an applied radiation force and resonant bubble oscillations. These responses can be measured via acoustic scattering, by direct imaging, or through light scattering.^146−148^

**Figure 11:**
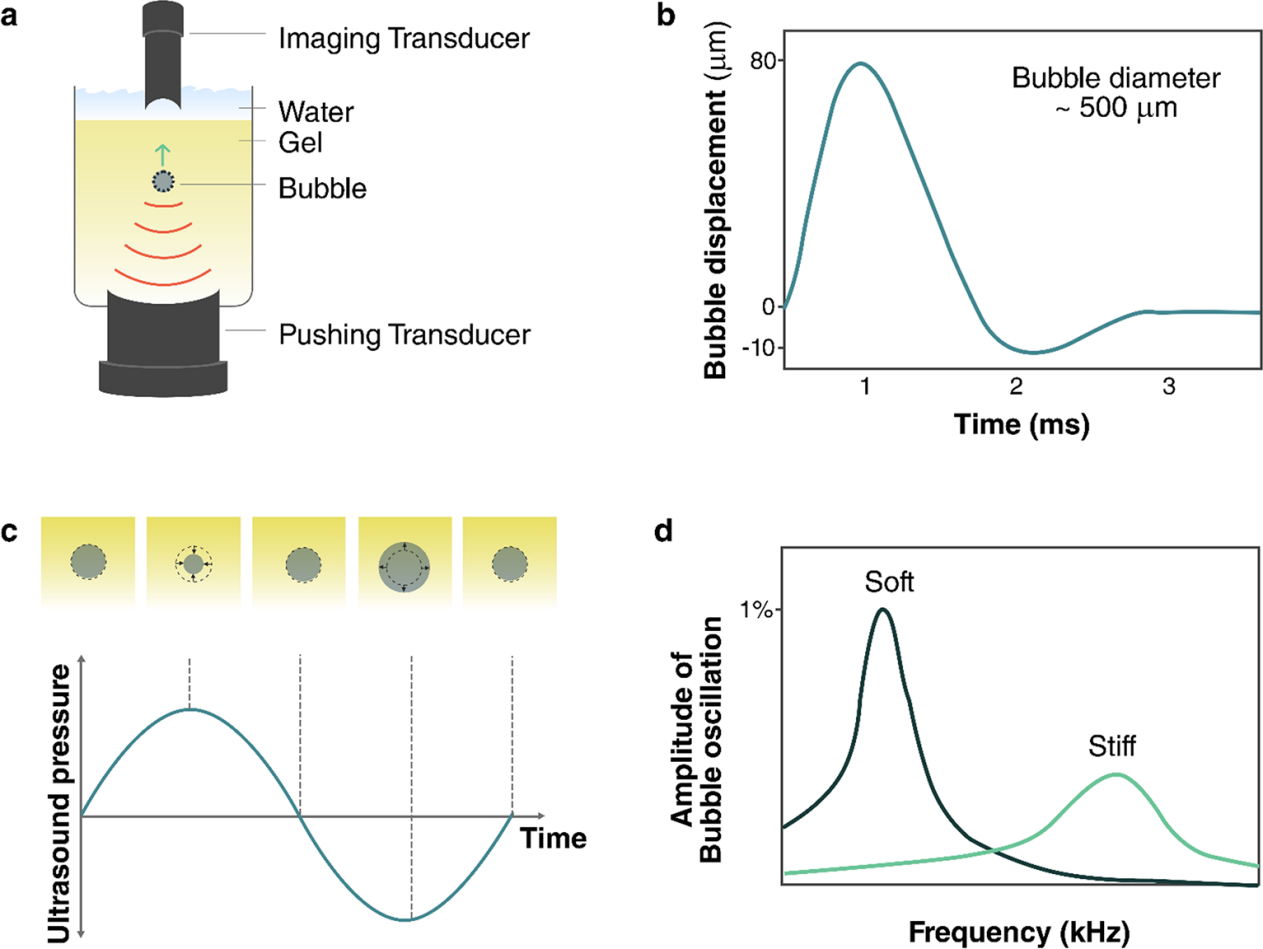
Microbubbles can be used to measure the mechanical properties of a material. Schemes for two acoustic schemes: (a) short ultrasound pulses push a bubble via the acoustic radiation force and (b) tracking its position over time reveals rheological and elastic properties of the medium. (c) A low amplitude ultrasound wave drives bubble oscillations, and (d) a shift in resonance is an indicator of a changing shear modulus or density.

In the bubble displacement technique, a short pulse of ultrasound pushes the bubble through a medium via the acoustic radiation force. The bubble’s change in position over time then reveals rheological and elastic properties of the medium (see Figure 11a,b).^149−151^ Erpelding et al.^149^ used this technique to measure the viscoelastic properties of gel phantoms remotely using ultrasound. Bubbles were generated in a phantom using laser-induced optical breakdown, and a two-element confocal ultrasound transducer targeted the bubbles. The outer element (driven at 1.5 MHz) provided the ARF, and the inner element periodically probed the bubble position via echolocation with short pulses centered at 7.44 MHz. The operating frequencies were higher than the bubble’s resonant frequency to minimize radial oscillations that could affect the motion. After correcting for differences based on bubble sizes, the authors could show that the displacement as a function of time is a measure for the Young’s modulus of the surrounding medium. Further validation of the bubble-based mechanical sensing techniques could be performed using elastography.^152^

In the oscillating bubble approach, a low-amplitude ultrasound wave is used to drive bubble oscillations. By scanning the drive frequency, the frequency-dependent vibration amplitudes of the bubble can be measured and resonant oscillations can be identified by a large response (see Figure 11c,d). Alekseev and Rybak^153^ used shifts in the resonance frequency for a known bubble size as an indicator of changing shear modulus or density within a medium.^153^

The resonance-based method can provide material information at much higher shear rates (up to 10^6^ s^–1^) than conventional techniques such as shear rheology. Jamburidze et al.^154^ experimentally characterized the resonant behavior of isolated microbubbles (100–200 μm diameter) embedded in agarose gels, which were excited by ultrasound between 10 and 50 kHz and with a small sound pressure amplitude of <1 kPa to remain in the linear regime. Observing the bubbles with high-speed video microscopy, the authors found that the resonance frequency increased linearly with the shear modulus of the gel, across a range of *G* = 7–256 kPa. These shear moduli were up to 5 times larger than values obtained from a rheometer at 1 Hz, revealing distinct material properties experienced at high shear rates.

One difficulty with the oscillating bubble measurements is that, at low acoustic pressures (e.g., 20 kPa), the small radial displacements (<30 nm) are challenging to observe using imaging techniques. Much smaller displacements, down to 10 pm, can be measured at high bandwidths using laser Doppler velocimetry, as demonstrated by Zhang et al.^155^ when measuring the response of submicrometer gas vesicles. A more economical alternative is to use an ultrasound imaging transducer and record the bubble’s acoustic scattering echo as an indicator of its vibration amplitude. A shift in resonance frequency and the appearance of higher harmonics can directly be seen in the acoustically measured scattering spectrum.^156,157^

Variations of these techniques have been explored to provide material information in different contexts. Using both bubble oscillations and ARF-based displacements, Saint-Michel and Garbin^158^ measured the viscoelastic properties of a yield-stress liquid (Carbopol gel). In addition to fluidlike systems, bubbles can also be embedded in soft solids as sensing elements. Lanoy et al.^159^ introduced a method to measure the complex shear modulus of soft silicones (e.g., PDMS) by including a layer of bubbles inside the material. The acoustic transmission spectrum was measured and fitted with an analytical model to calculate the shear modulus. This method shows promise to continuously monitor the aging process of a smart material.

#### 3.3.2 Piezo-Based Sensing and Stimulation

Ultrasound can also be used to probe the electrical properties of a region inside a material. As mentioned in section 2.3.1, the piezeoelectric effect couples mechanical strain to electrical charge. This section covers the detection and generation of electric signals by small-sized piezoelectric crystals that are excited remotely via ultrasound.

**Figure 12:**
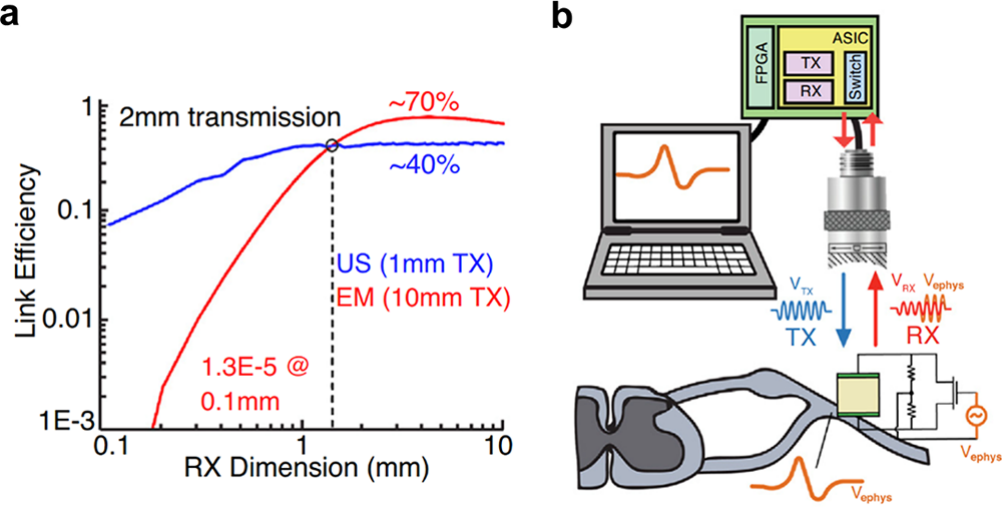
Operating principles for neural dust. (a) Link efficiency for ultrasound (US) and electromagnetic (EM) coupling through biological tissue versus dimension of the receiver (RX). Ultrasonic links outperform EM links below 1 mm. (b) Schematic for remote sensing of physiological action potentials. The local electric potential influences the backscattering signal of the piezo element. This information can be recovered remotely via ultrasonic imaging. Reproduced with permission from ref (160). Copyright 2018 Elsevier.

“Neural dust” is a concept name for wireless brain–machine interfaces based on this principle.^160−162^ Tiny motes consisting of a single piezoelectric particle with reduced electronics are spread throughout a volume, e.g., in brain tissue. The use of ultrasound for powering and communication allows these motes to be implanted centimeter deep into tissue. Further, the link efficiency scales more favorably with ultrasound compared to wireless electromagnetic devices when the characteristic dimensions are reduced (see Figure 12a).

The concept of neural dust is explained by the schematic in Figure 12b. Each piezo crystal is connected to a compact electrical network including a field effect transistor (FET). The FET couples the electrical load impedance of the circuit to the surrounding electric potential, which affects the elastic behavior of the crystal. When ultrasound is focused onto the neural dust mote, the backscattered echo is modulated by the local electric field, revealing information about action potentials and enabling wireless probing of neuronal activity. By using ultrasound imaging transducers, electric states of dust motes in potentially many different places can be monitored in parallel. *In vivo* electromyograms and electroneurograms have been remotely recorded using a single mote implanted in a rat.^162^ The smallest mote to date has been reported by Shi et al.,^163^ which is capable of measuring temperature in just a 0.1 mm^3^ package. The authors demonstrated the device *in vivo* implanted in brain and muscle tissue of mice.^163^

Stim dust is complementary to neural dust, expanding the concept toward stimulation of nerves.^164^ Nerves and muscles can be excited remotely using the direct piezoelectric effect, which converts mechanical (acoustic) energy into electrical energy. A major hurdle, however, is to provide a controlled current output via the remote power link. On the one hand, high-frequency acoustic waves (typically megahertz) should be used in order to focus all of the energy efficiently into a small region around the sub-millimeter-scale device. On the other hand, nerves respond to transient action potentials with millisecond time scales, which would be better driven by low-kilohertz acoustic waves. To bridge the temporal gap between the ultrasonic and biological worlds, Piech et al.^164^ showed that electronic rectifying circuits can be used, converting the megahertz-frequency ultrasonic energy into lower-frequency signals for nerve stimulation. The authors successfully combined a piezoceramic element, energy-storage capacitor, and integrated circuit into a 1.7 mm^3^ small device and demonstrated it *in vivo* mounted on the sciatic nerve of anesthesized rats. The device was capable of delivering 50–400 μA pulse amplitudes, pulse widths of up to 392 μs, and pulse repetition frequencies up to 5 kHz. This was sufficient to excite compound action potentials and cause twitches in the rat’s muscles. In these experiments the ultrasound field had a derated *I*_SPPA_ = 692 mW cm^–2^ and a mechanical index MI = 0.11, both below the safety limits set by the FDA.^56^

All of these devices use active elements made of lead-based piezoceramics, which are toxic to humans and thus limit biocompatibility. To realize the potential of implantable sensing and stimulation devices, more work is needed to improve the efficiency of biocompatible piezo materials.

#### 3.3.3 *In Vivo* Sensing of Biomolecular and Cellular Processes

*In vivo* imaging techniques to monitor biological and cellular processes are highly desirable but challenging to realize. Established optical methods for noninvasive imaging of biological tissues are limited to low penetration depths due to strong scattering. In contrast, the low attenuation of ultrasound gives it an advantage to visualize biomolecular events *in vivo*.

Ultrasound molecular imaging can be achieved by monitoring the change in ultrasound scattering intensity by microbubbles that are injected intravenously.^165^ The concept is based on engineered microbubbles, whose lipid shell is covered with ligands that can selectively bind to cells on the surface of blood vessels.^54^ The microbubbles have a typical size of 1–10 μm, which permits circulation throughout the vasculature and enables targeting regions inside capillaries (diameter < 10 μm). This size range also provides good acoustic contrast, as is also exploited in microbubbles as commercial ultrasonic contrast agents. The use of smaller bubbles to probe extravascular structures has not been fully explored, hampered by their much weaker scattering response at the relevant medical ultrasonic frequencies.^54^ Recent developments, however, show promise to extend ultrasound molecular imaging to smaller bubble sizes. For instance, Jafari Sojahrood et al.^166^ reported fabrication of shell-stabilized nanobubbles with a precisely controllable acoustic response. It has been shown that the scattering response of encapsulated microbubbles behaves nonlinearly and is strongly amplified beyond a threshold excitation pressure *p*_t_. This threshold depends on the elastic properties of the shell and can be tuned to maximize signal response with minimal bubble collapse. In this study the authors used propylene glycol as a membrane softener or glycerol as a membrane stiffener and fabricated bubbles of 200 nm mean diameter and varying thresholds in the range 120–710 kPa.

An alternative to manufactured micro- or nanobubbles is gas vesicles. These are “gas-filled compartments with a protein-shell with typical widths of 45–250 nm and lengths of 100–600 nm.”^167^ Their shell consists of proteins known as Gvp proteins, with GvpA being the main constituent.^168^ The difference in hydrophobicity and hydrophilicity within the protein structure allows the permeation of gas while excluding liquid water. This enhances the stability of vesicles in comparison to an uncoated nanobubble, which quickly dissolves because of the high Laplace pressure.

Gas vesicles show promise as a tool for molecular imaging using ultrasound. Bourdeau et al.^169^ created a so-called acoustic reporter gene (ARG) that expresses the proteins necessary to stabilize the gas vesicles. As a proof of concept, the authors demonstrated the use of ARG to locate two different types of genetically engineered bacteria inside the gastrointestinal tract of a mouse.^169^ More recently it has been shown that ARGs can be used to sense enzyme activity in probiotic bacteria.^170^

**Figure 13:**
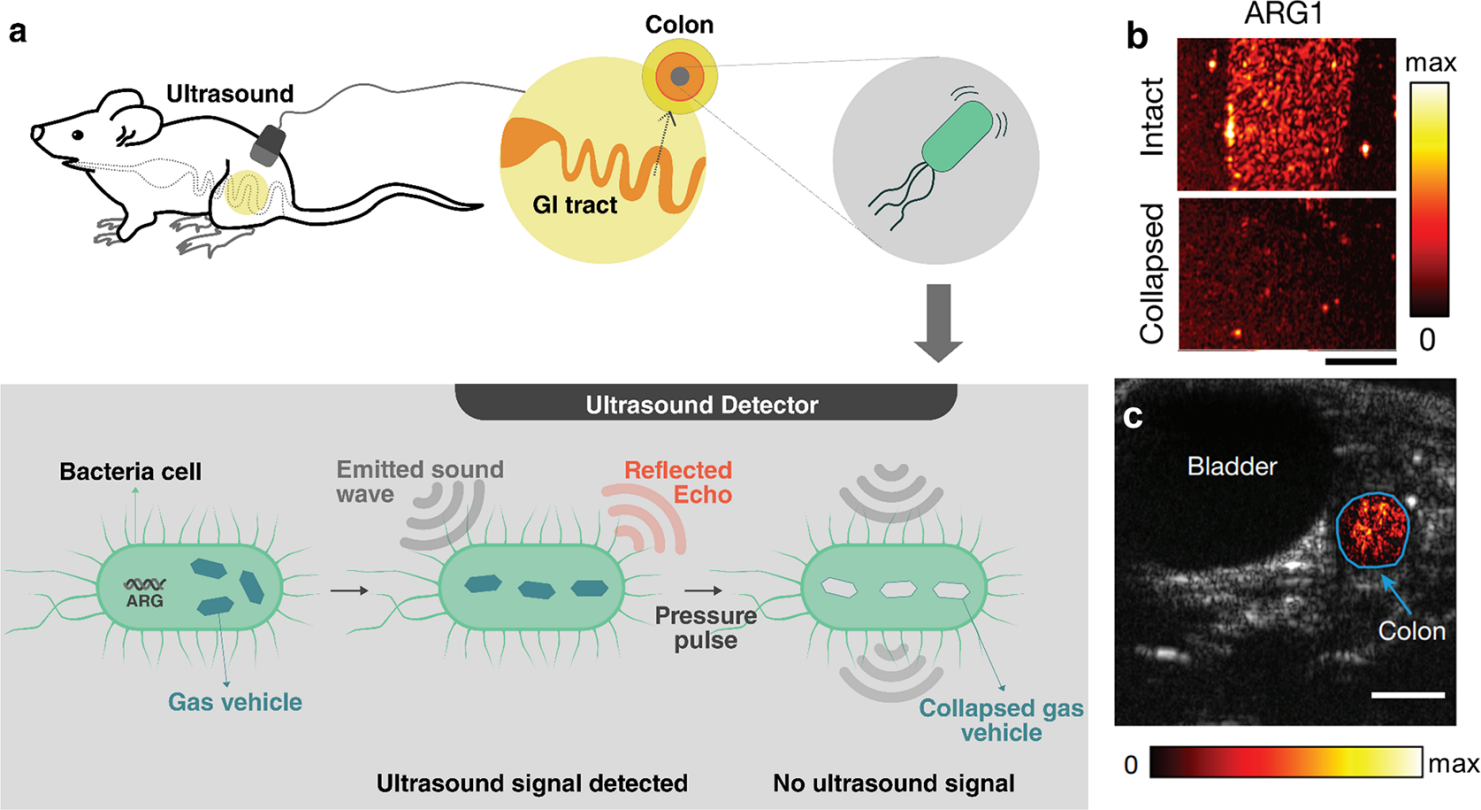
(a) Sensing of gas vesicles inside the gastrointestinal (GI) tract of a mouse: expression of the acoustic reporter gene (ARG) in probiotic bacteria generates gas vesicles that show ultrasound contrast. (b) Ultrasound images of gel phantoms containing *E. coli* bacteria expressing arg1 before and after collapse of gas vesicles (scale bar 2 mm). (c) Transverse ultrasound image of a mouse colon containing *E. coli* bacteria expressing arg1 proximal to the colon wall (scale bar 2.5 mm). Panels b and c adapted with permission from ref (169). Copyright 2018 Springer Nature.

Gas vesicles can be remotely observed with ultrasound scattering, as shown in Figure 13a. In general, gas vesicles give rise to a bright backscattered contrast (echogenicity) in ultrasound imaging. The scattered intensity depends on the gas vesicle size,^169^ which can be tailored by genetically engineering the coating protein composition,^171^ or by changing the bacterial species that produces the vesicles.^167,169^ In general, smaller gas vesicles exhibit low echogenicity.^169^ In addition to linear scattering, nonlinearly scattered signals can also be used to accurately localize gas vesicles.^172,173^ The nonlinear response also depends strongly on the strength and composition of the protein shell, which can be controlled by changing the producing species,^167^ or direct genetic engineering.^171^ For example, irradiated with 6 MHz pulses (peak amplitude 98 kPa) the gas vesicles produced by the microorganism *Halobacterium* NRC-1 produced second- and third-harmonic signals at 12 and 18 MHz, but vesicles produced in *Anabaena flos-aquae* did not.^167^

The hydrostatic collapse pressure of the vesicle structure can also be used to confirm the presence of distinct species by comparing images before and after collapse (see Figure 13b). Above a certain confining pressure, the protein shell of the vesicles buckles and the gas can diffuse away, eliminating any echogenicity. The critical hydrostatic collapse pressures range from 40 kPa to over 700 kPa depending on bacterial species.^174,175^ However, these values do not directly translate to acoustic collapse pressures. For example, *Halobacterium salinarum* collapsed at acoustic pressure amplitudes 9 times higher than the critical pressure observed under quasi-hydrostatic conditions.^176^ Since the collapse pressure is controlled by the vesicle protein composition, acoustic monitoring of collapse can be used to distinguish different vesicle-carrying species.^167,169,171^ Vesicles can therefore be differentiated based on absolute echogenicity differences (linear and nonlinear) or alternatively using the echogenicity difference before and after vesicle collapse. For ultrasonic observation of gas vesicles, the most critical factors are thus the size and protein composition of the stabilizing shell, both of which can be adjusted by appropriate species selection and genetic engineering.

The functionality of gas vesicles continues to grow due to continuing advances in genetic engineering. For example, by engineering the vesicle-producing ARG, the gas vesicles’ responsiveness to ultrasound can be tuned (e.g., the collapse pressure or the scattering strength).^171^ By using properly designed ARGs in one setting, the differences in acoustic response can be used to monitor multiple different biological processes simultaneously. Additionally, expressing the ARG in bacterial hosts can enable targeted cavitation of the gas vesicles. This has been shown to complement bacteriotherapy with high-intensity focused ultrasound (HIFU) as a theranostic approach to treat breast cancer. The ARG was expressed in *E. coli*, which specifically targeted and colonized the tumor site inhibiting the tumor growth. The gas vesicles further acted as nuclei for cavitation during HIFU ablation of the tumor.^177^

In the future it will be of interest to express ARGs not only in bacterial hosts, but also in mammalian cells, as was recently accomplished by Farhadi et al.^178^ However, the ARG currently requires much longer time to express the gas vesicles in mammalian hosts (days) than in bacterial host (hours). A review that explores further directions and perspectives of ultrasound technologies for neuroimaging and neuromodulation has been written by Rabut et al.^179^

### 3.4 Payload Transport and Delivery

#### 3.4.1 Payload Transport and Release

Ultrasound can be used to control the spatial and temporal release of substances. Its tremendous potential for remote activation garnered with its biocompatibility and low attenuation in tissue has steadily motivated the field of smart drug delivery systems.^55,180,181^ The state of the art in ultrasound-triggered payload delivery is based on encapsulated microbubbles. Current efforts can be grouped into four broad classes, based on the specific carrier technologies that they use: microbubbles (section 3.4.1.1), phase-change nanodroplets (section 3.4.1.2), nanocarriers (section 3.4.1.3), and emerging carriers (section 3.4.1.4).

##### 3.4.1.1 Microbubbles

Microbubbles play a central role in efforts to transport and release a chemical or nanoparticle payload upon ultrasound exposure. Microbubbles have been studied for more than 30 years, initially as ultrasound contrast agents and more recently as targeted drug carriers operated using medically relevant ultrasound.^54,55,180−185^ This section aims to cover selected examples from this highly active research field, presenting case studies to illustrate possibilities and limitations when developing smart systems that require the transport and release of chemical payloads. Readers interested in the use of microbubbles as ultrasound contrast agents and as drug delivery vehicles should consult recent comprehensive reviews.^54,55,181,183^

**Figure 14:**
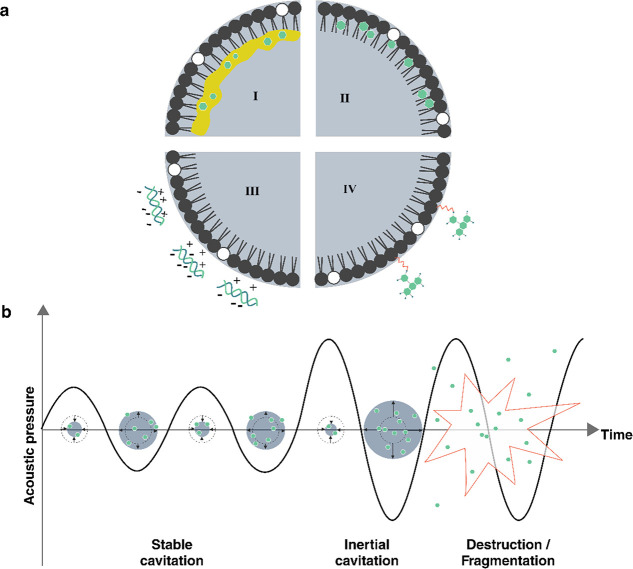
Microbubbles can be used to transport and release a payload upon ultrasound exposure. (a) Scheme showing the different ways to accommodate the payload in the microbubble structure: (I) using an oil layer, (II) within the shell, (III) via electrostatic binding, and (IV) direct linkage to the surface. (b) Microbubble response to different ultrasound regimes.

When irradiated with ultrasound, bubbles display volumetric oscillations that are responsible for mechanical, thermal, and chemical effects.^55^ The extent of these effects is determined by the surrounding medium and the microbubble structure, namely the type of gas enclosed as well as the composition of the stabilizing shell. In the context of drug delivery, the shells are typically composed of lipids enclosing perfluorocarbon gas, which have low solubility in water. The payload can be included by dissolving it in an oil layer inside or within the shell, by electrostatic binding to the outer surface, or by directly linking the surface to molecules or nanocarriers,^186^ as illustrated in Figure 14a. Alternatively, shells can be made of proteins, surfactants, or polymers,^187^ while other gases such as oxygen can also be enclosed, although the stability becomes a challenge.

Microbubbles can be used either directly as payload carriers or indirectly to enhance chemical transport, e.g., through cavitation induced sonoporation (see section 3.4.2). When microbubbles are used as carriers, an acoustic pressure beyond a critical amplitude leads to rupture and release of the shell fragments,^188^ as shown in Figure 14b. By this method the payload can travel hundreds of micrometers *in vivo*(189) and millimeters in gel media.^190^ The detailed mechanism of this delivery mechanism is not yet fully understood. For a recent review covering control of cavitation for drug delivery, see ref (55). For indirect delivery techniques, irradiating cells with low-intensity ultrasound in the presence of exogenous microbubbles has been shown to enhance permeation into the cells.^191,192^

Microbubbles have been studied for targeted delivery of chemotherapy drugs,^193,194^ for gene delivery,^195,196^ and to open the blood–brain barrier.^197,198^ Extension of microbubble techniques and functionalities in different contexts has also revealed application-specific limitations. For example, to treat hypoxic tumors microbubbles carrying oxygen are preferred, but their low lifetime leads to a short circulation (<5 min) and hence low targeting efficiency when administered systemically.^199^ To overcome this limitation, different targeting techniques can be employed. For instance, microbubbles can be covered with ligands that specifically bind to a target site. The targeting in this case can be further enhanced by using acoustic radiation force to increase the microbubble concentration near the target.^200^ Alternately, the microbubble shell can be functionalized with superparamagnetic iron oxide nanoparticles and aggregated at the target site using magnetic fields.^193,201,202^ One study used a combination of magnetic and acoustic fields,^193^ which were realized with a focused ultrasound transducer and a permanent magnet combined in a single device. The fixed alignment made it possible to aggregate and excite microbubbles with intense ultrasound (1 MHz, 3 W cm^–2^) and keep them in focus. The authors found a significantly enhanced reduction in tumor size within the first 8 days that was not observed using only one of the two fields independently.^193^

Microbubbles can also be used to disperse nanoparticles in complex media. Recently, Baresch and Garbin^203^ trapped nanoparticle-coated microbubbles with an acoustic vortex beam and released the nanoparticles using ultrasonic excitation from a second transducer. The experiments were conducted in agarose (shear modulus *G* ≈ 10 kPa). For bubbles excited close to resonance, large-amplitude nonspherical oscillations caused the particles to eject in multiple directions as plumes, propelling them multiple bubble diameters into the gel.

While microbubbles form the basis for many techniques in payload delivery, their size imposes difficulties in their use, especially *in vivo*. On the one hand, micrometer-scale gas bubbles are difficult to stabilize against diffusion, especially in the presence of ultrasound. On the other hand, micrometer-scale bubbles are too large to permeate into smaller areas of interest including extravascular structures such as tumors.^204^

##### 3.4.1.2 Phase-Change Nanodroplets

**Figure 15:**
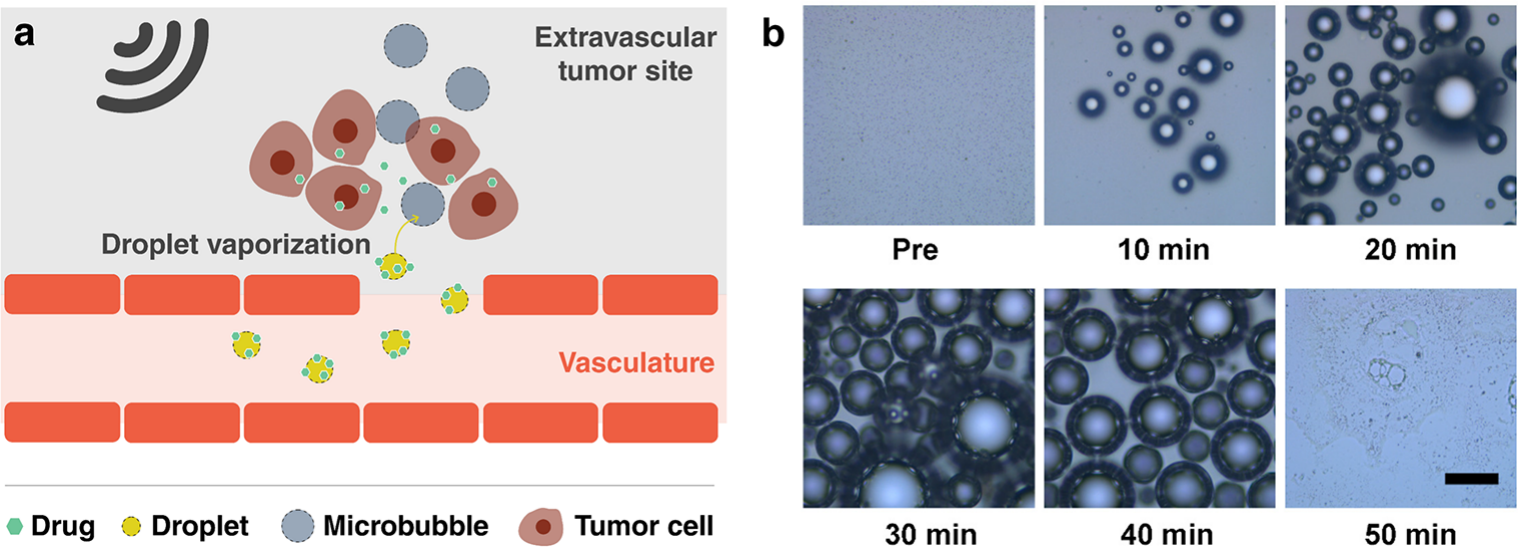
(a) Illustration of drug delivery through an extravascular barrier using phase-changeable nanodroplets. (b) Time-resolved microscope images of PFC nanodroplets before and after ultrasound exposure. Scale bar 50 μm. Reproduced from ref (205). Copyright 2019 American Chemical Society.

To address the current challenges of microbubbles, phase-change nanodroplets have emerged as alternative carriers and have been developed for improved stability, longevity, and extravasation.^206^ Nanodroplets are vesicles that contain a core of phase-changeable material and that can be decorated with functional drugs.^207^ Their size range (400–800 nm)^206^ is comparable to the gap between endothelial cells (380–780 nm),^208^ which can enhance uptake of drugs due to improved extravasation into tumor tissues (Figure 15a). Commonly used materials for the core are perfluorocarbons (PFCs), because they are nontoxic and have low solubility in water, improving the lifetime of the droplets.^209,210^ PFCs are volatile compounds: for example, the boiling temperature *T*_b_ of perfluoropentane (PFP) is 29 °C and that of perfluorohexane (PFH) is 56 °C.^211^ Thus, when the liquid droplet is exposed to physiological temperatures, it becomes metastable and readily transitions to the gas phase upon excitation with ultrasound.^212^ This process, known as acoustic droplet vaporization (ADV),^213,214^ causes a dramatic change in size. Depending on composition, nanosized liquid droplets with diameters of 200–300 nm can expand into 1–5 μm gas bubbles.^205,215^ The evolution of growing PFC gas bubbles after acoustic vaporization is shown in Figure 15b. While the nano liquid droplets provide transport stability in a small-scale carrier, the expansion process can be used for mechanical agitation and stronger ultrasonic contrast. These characteristics benefit diagnostic and therapeutic uses such as ultrasound imaging,^216−218^ drug delivery,^219−221^ BBB opening,^222^ and sonothrombolysis.^205,223,224^

While most demonstrations using PFC droplets have required high-intensity ultrasound, the nanodroplet design can be modified to trigger phase change at lower intensities. The use of materials with low boiling points (e.g., decafluorobutane (DFB), *T*_b_ ≈ −1.7 °C)^215^ is one approach to reduce the vaporization threshold intensity,^207,225,226^ approaching the safety limits for clinical ultrasound (see section 2.3.5). The vaporization threshold is further influenced by surface tension and droplet size.^227,228^

Different techniques have emerged for multistage payload release using PFC nanodroplets. Cao et al.^229^ demonstrated that different release stages could be triggered at different ultrasound intensities by properly designing the nanobubble shell. They produced two different droplets made of different shell materials: either lipid based (softer) or PLGA based (harder). The droplets could be vaporized with 1 MHz ultrasound at electrical driving powers of 3 and 8 W, respectively. The treatment then proceeded in two stages. First, the soft-shelled nanodroplets generated small pores, which enabled the hard-shelled droplets to diffuse deeper into the tissue before being vaporized and releasing the drug doxorubicin.^229^ Aliabouzar et al.^230^ demonstrated that nanodroplets made with different PFC cores could be triggered at different frequencies. They created two kinds of nanodrops containing either perfluorohexane or perfluorooctane along with a molecular payload. While both droplet types could be vaporized at 2.5 MHz, only the perfluorohexane droplets vaporized at 8.6 MHz, allowing them to be activated first using the high-frequency excitation.

Acoustic droplet vaporization can also be used as a microscale ballistic tool to propel particles, a concept which has been demonstrated by Soto et al.^231^ Hollow tubes were filled with silica microspheres (diameter 1 μm) or fluorescent polystyrene spheres (diameter 100 nm) as well as PFC embedded in a gel matrix stabilizer. An acoustic pulse triggered vaporization of the PFC, ejecting the particles. The nanoparticles were found to travel 17.5 μm into a gelatin phantom.

Phase-change nanodroplets show enhanced performances over microbubbles for payload delivery through small regions. However, the triggering thresholds of nanodroplets are based on a complex interplay of composition and ultrasound parameters, and the resulting effects of vaporization at the target site are not yet fully understood. For future clinical applications it is important to predict and control the behavior of ultrasound on the vaporization and its effect in tissue.^206^

##### 3.4.1.3 Nanocarriers

Nanocarriers present a third option for the ultrasound-triggered release of payloads.^232^ They comprise different inorganic and organic particles with sizes up to several hundred nanometers, and their size enables them to access hard-to-reach places.^233^

**Figure 16:**
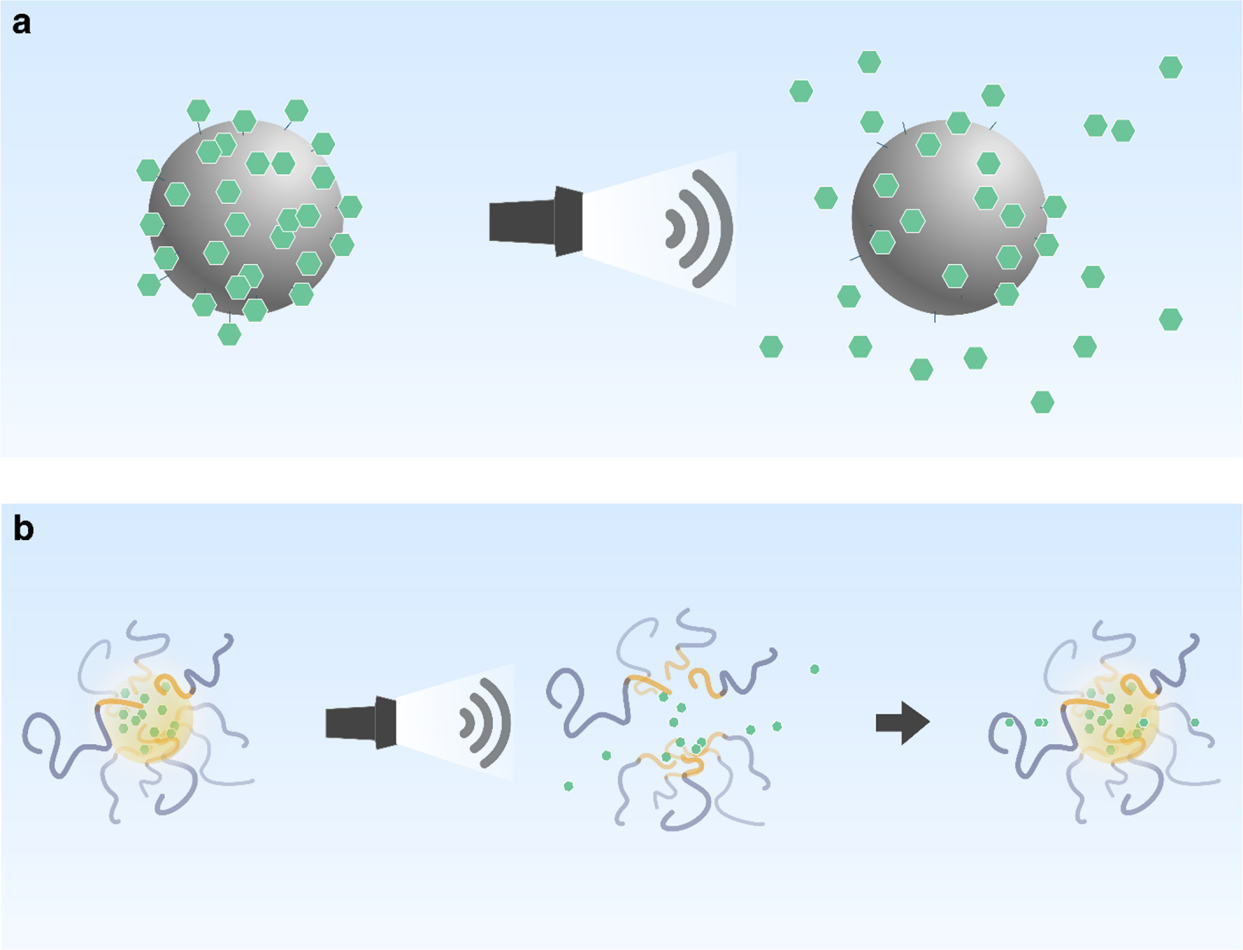
Mechanism for payload release of nanocarriers. (a) Irreversible and (b) reversible releases of payload from nanocarriers. On the left in (a) and (b) are the initial states of the nanocarriers with the payload on the surface and encapsulated inside, respectively. Irradiation of the nanocarriers with ultrasound (20–90 kHz) induces cavitation, where the emitted shock wave and temperature increase (a) destroy the nanocarrier or (b) temporarily open the structure for the release of the payload.

Most inorganic carriers can transport a payload either adsorbed or conjugated to the surface. Examples include mesoporous silica particles (MSNPs), gold nanoparticles, superparamagnetic iron oxide nanoparticles (SPIONs), and carbon nanotubes (CNT).^185^ When irradiated with low-frequency ultrasound (20–90 kHz), the payload is irreversibly detached by a cavitation process, as shown in Figure 16a. Cavitation is caused by nucleating freely dissolved gas or interfacial gaseous voids located on the rough surface of the particles.^234^ MSNPs and CNTs can also be used as air-containing nanocarriers.^235,236^ The high hydrophobicity of CNTs allows them to retain air stably inside their hollow structure, which can be used to enhance cavitation or as contrast by reflecting for high-frequency ultrasound.^237^ Cavitation effects can be further enhanced with external stimulation. For example, it has been shown that irradiating gold nanoparticles with intense light pulses generates bubbles whose presence lowers the ultrasonic pressure threshold for cavitation.^234,238^

In situations where surface conjugation is not straightforward, self-assembled organic nanocarriers such as liposomes, nanoemulsions, polymeric micelles, or polymersomes can be used to encapsulate the cargo within their structures. Unlike surface-loaded nanoparticles, their response to ultrasound depends on specific characteristics of the self-assembled structure. For example, the temperature increase caused by focused ultrasound (1.1 MHz) in hyperthermia treatment also enhances the permeability of liposomes and hence the release of a payload.^239^ Irradiation at lower frequencies but higher amplitudes generates shear stresses that can rupture vesicles. However, the ruptured liposomes will readily re-form multiple smaller vesicles with the same total surface area (assuming no loss of phospholipids).^180,240^ Hence, this mechanism involves a partial release of cargo from the liposomes.^180^ Similarly, polymersomes also show a size reduction proportional to the duration and power of the applied ultrasound (20–40 kHz, 0–180 W).^241,242^ In contrast, polymeric micelles temporarily release the payload when irradiated with low-frequency ultrasound (20–90 kHz), but then reencapsulate most of the cargo after the ultrasound exposure has stopped, suggesting a reversible release when compared with surface loaded nanocarriers, liposomes, and nanoemulsions (see Figure 16b).^243^ This behavior implies that polymeric micelles could be used for longer term applications that require release over multiple exposures to ultrasound.^244^ This so-called “reversible mechanism” for payload release requires higher power densities at higher frequencies.^245^ The onset of release occurs above a threshold ultrasound intensity (0.3 W cm^–2^ at 70 kHz)^246^ and pulse length (0.1 s at 20 kHz with intensity 58 mW cm^–2^).^247^ Recently, it has been proposed that the dominant factors in the ultrasound response of self-assembled structures are the solvent type and the temperature at which the structures are self-assembled.^248^ In this study, block copolymer micelles and vesicles (polymersomes) were irradiated with low-frequency ultrasound (20 kHz, 37.5 W, 3 min). Improved ultrasonic bursting and reassembly were observed when the temperature at which the polymer chains were self-assembled is close to the glass transition temperature of the hydrophobic segment. This principle was applied by the same authors to fabricate pH–ultrasound responsive polymersomes to release the chemotherapeutic drug doxorubicin. The polymersomes were designed to respond to the slightly more acidic pH of the tumor microenvironment by initiating the release with ultrasound irradiation (20 kHz, 45 W).^242^

At high frequencies, polymeric micelles irreversibly release payloads via bond breakage of ultrasound responsive groups within the structure of the amphiphilic units. This was demonstrated by using high-intensity focused ultrasound at frequencies close to 1 MHz.^249,250^ Bond breakage occurred at the labile bond sites, which are sensitive to thermal and mechanical effects induced by ultrasound (see section 3.5.2).

**Figure 17:**
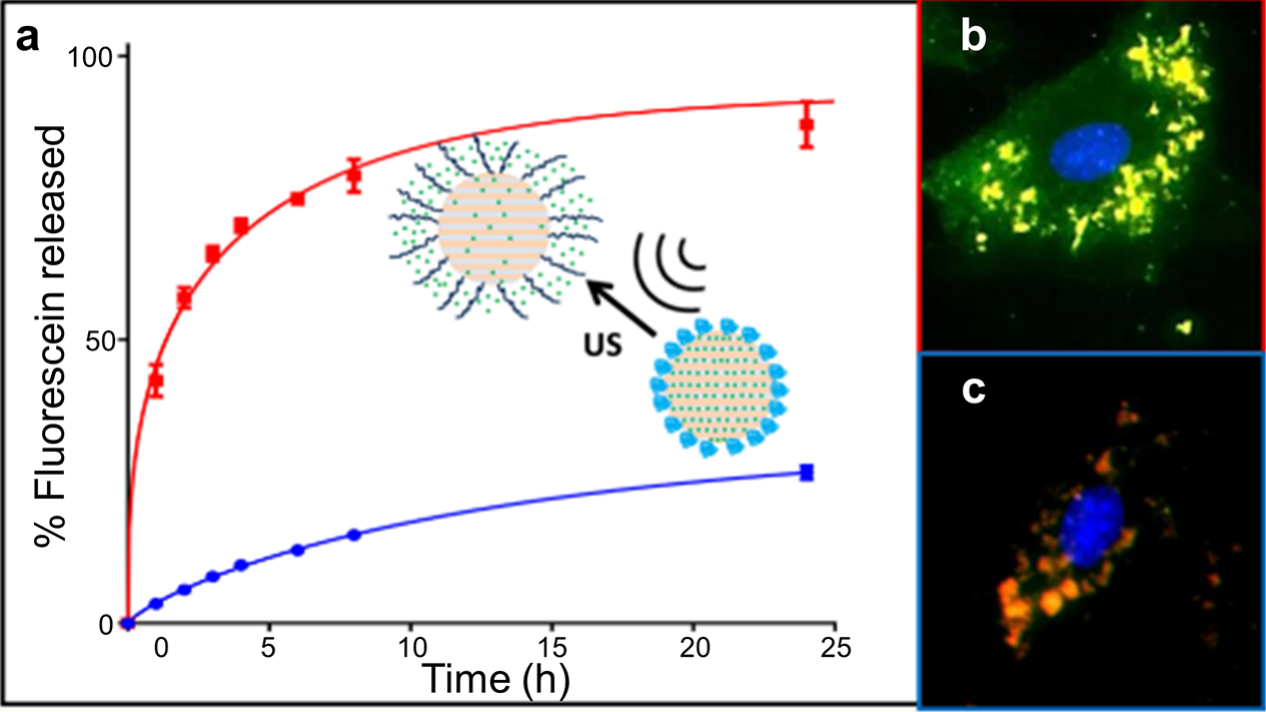
(a) Plot of the release of fluorescein in solution from a mesoporous silica nanoparticle (MSNP) grafted with a HIFU sensitive block copolymer (10 min and 1.3 MHz, 100 W). (b, c) Fluorescence microscopic images of cells incubated with rhodamine B labeled MSNPs–polymer with fluorescein shown respectively before and after ultrasound exposure. Adapted from ref (251). Copyright 2015 American Chemical Society.

Many more options for ultrasound-responsive nanocarriers emerge from combinations of the examples above. The combination of liposomes with microbubbles is a common approach^185^ that has recently been used to trigger enzymatic gelation.^252^ The liposomes contained calcium ions that were ultrasonically released in the presence of the enzyme transglutaminase, forming fibrinogen hydrogels through covalent intermolecular cross-linking. A less common, yet interesting, example is the combination of MSNPs with ultrasound-sensitive block copolymers. For example, by grafting block copolymers with ultrasound sensitive bonds, similar to polymeric micelles, a gating effect over the pores of the MSNP can be used to trigger the release of a payload, as shown in Figure 17.^251^ This study used high-intensity focused ultrasound (HIFU; here 1.3 MHz, 100 W) to break labile bonds and switch the polymer chain to a hydrophobic state, where its structure is expanded, allowing the payload to be released. These examples demonstrated new ways to release payload from the nanocarriers. Many current systems rely on surface immobilization, which requires a surface chemistry that can couple the payload to the carrier. A solution to this problem can lie in supramolecular host–guest complexes, where the reversible binding may be disrupted by ultrasound,^253,254^ although the inclusion of the supramolecular host to the carrier might still be a challenge. Lastly, inorganic vesicles made of self-assembled Au–MnO Janus particles are an example of a new functionality established through the material selection of inorganic carriers. These vesicles disassemble following ultrasound irradiation and permeate deep through liver tumors to generate radical oxygen species after glutathione triggered MnO degradation.^255^

##### 3.4.1.4 Emerging Carriers

Emerging carriers are providing promising new directions and capabilities for payload delivery. Although these techniques are still in their infancy, we summarize this early work here to provide insight into new directions for the field.

An alternative carrier to conventional microbubbles has recently emerged in the form of Pickering-stabilized antibubbles.^256,257^ Antibubbles consist of a liquid core surrounded by a thin gas layer that separates the core from the surrounding fluid. Recently it has been shown that the liquid core causes antibubbles to oscillate asymmetrically, which gives rise to higher harmonics with nonlinear scattering strengths comparable to or higher than those for conventional microbubble contrast agents.^258,259^ The nonlinear radius oscillations may also provide a mechanism to more easily burst the antibubble and deliver a payload from the core, avoiding the surface modifications required by microbubble techniques. Further functionalities can be added to antibubbles by appropriate payload selection. For instance, magnetically responsive antibubbles were produced by dispersing Fe_3_O_4_ particles in the liquid core.^260^ While still an emerging topic, antibubbles offer new potential pathways to realize ultrasound triggered payload release.

In contrast to microbubble- and nanocarrier-based systems, hydrogel carriers enable the stepwise release of a payload. Most of the systems mentioned above only permit one time release triggered by ultrasound or the gradual release over longer time periods. However, there are scenarios in which it is desirable to release the payload over multiple large doses at arbitrary times. Such systems have been described as permitting digital drug release.^261,262^ This concept has been demonstrated using hydrogels.^261−268^ Huebsch et al.^261^ studied biocompatible injectable alginate hydrogels for on-demand release of the chemotherapeutic drug mitoxantrone. They showed that ultrasound pulses (20 kHz, 9.6 mW cm^–2^, pulse length 5 min, pulse repetition frequency (PRF) 1 h^–1^) disrupt the ionically cross-linked polymer network, releasing the mitoxantron. Once the ultrasound stops, the self-healing of the hydrogel prohibits a further release of the chemotherapeutic. Recently, it has been shown that ultrasonic exposures needed to generate significant therapeutic deliveries from calcium-cross-linked hydrogels also generated high levels of gel heating and erosion—an effect that can be mitigated with pulsed ultrasound.^267^

#### 3.4.2 Opening Biological Barriers

Ultrasound can open biological barriers (e.g., cell, blood–brain) and make them more permeable for the delivery of therapeutic payloads.

**Figure 18:**
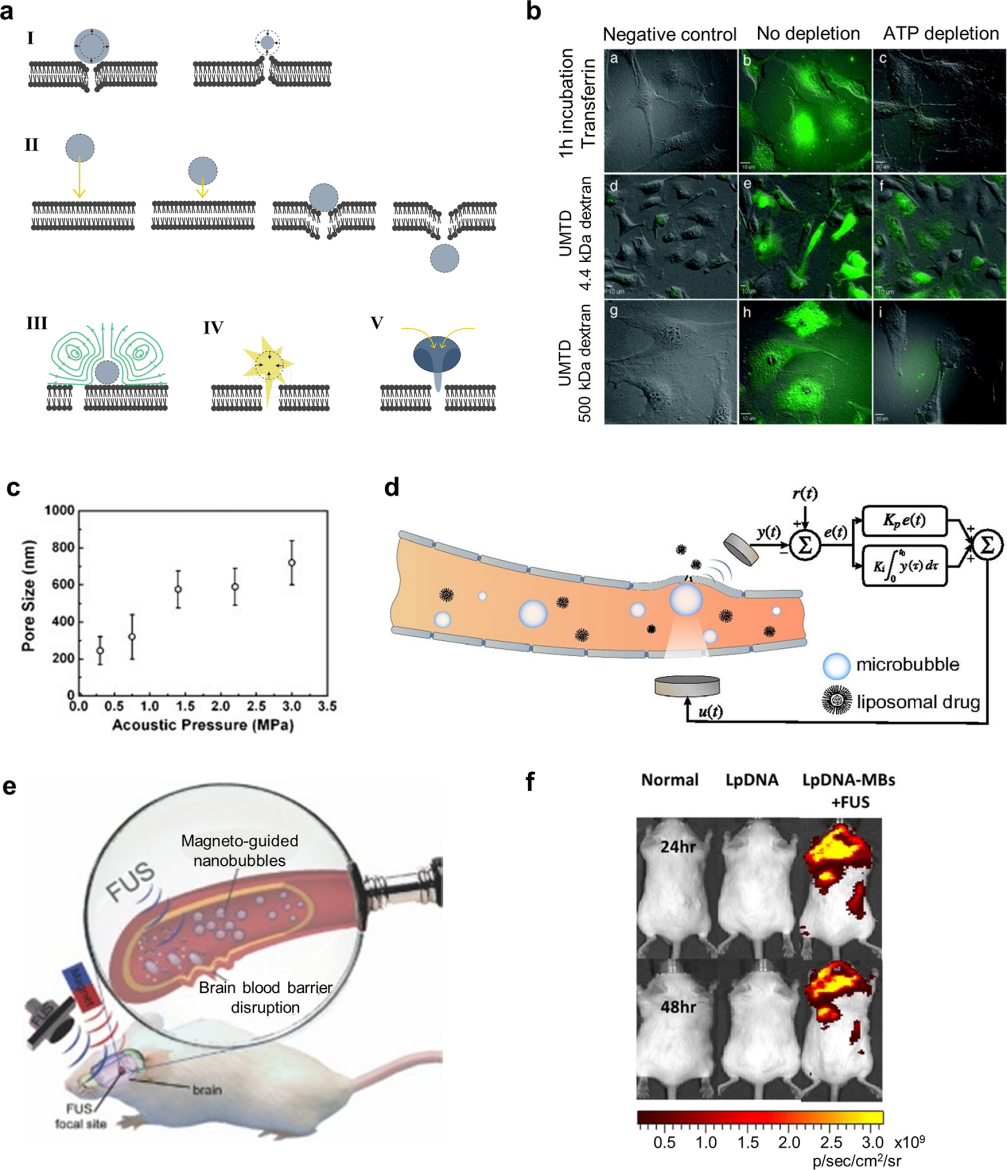
(a) Mechanism of sonoporation by ultrasound-induced microbubble cavitation ((I–III) stable cavitation-induced sonoporation; (IV, V) inertial cavitation-induced sonoporation). Adapted with permission from ref (269). Copyright 2014 Elsevier. (b) Cellular uptake of transferrin and fluorescent dextrans of different molecular weights (4.4 and 500 kDa) by endocytosis (no depletion) and diffusional process (ATP depletion) under ultrasonic radiation for 30 s (1 MHz ultrasound, 20 Hz pulse repetition rate, 0.22 MPa peak negative pressure). Reproduced with permission from ref (272). Copyright 2009 Wolters Kluwer Health. (c) Pore size created by sonoporation as a function of ultrasound pressure. Reproduced with permission from ref (277). Copyright 2010 Elsevier. (d) Focused ultrasound induced microbubble cavitation, locally opening the blood–brain barrier for drug transmission. Cavitation can be monitored by an acoustic detector and applied with closed-loop control. Reproduced from ref (284). CC BY 4.0. (e) The nanobubbles can be labeled with paramagnetic particles, enabling a magnetically guided blood–brain barrier opening process. Reproduced with permission from ref (290). Copyright 2014 John Wiley and Sons. (f) *In vivo* bioluminescent imaging verifies the ultrasound activated microbubbles enhancing gene delivery across the blood–brain barrier. LpDNA, liposome-containing pDNA, MBs, microbubbles, FUS, focused ultrasound. Reproduced with permission from ref (289). Copyright 2016 Elsevier.

Sonoporation (the opening of the cell membrane with ultrasound) is triggered by cavitation and associated bubble-driven streaming in the vicinity of a cell membrane.^269^ The resulting large shear stresses deform the cells and form pores,^270,271^ which leads to endocytosis, opening the cell membrane such that molecules can passively diffuse into the cell^272^ (see Figure 18a). Similar effects have also been observed for enhancing delivery through skin.^273,274^ Sonoporation has been confirmed by real-time confocal microscopy that the ultrasound-stimulated bubble oscillation generates the shear stress above the threshold of pore formation on the cell membrane.^275^ Recently, sonoporation has also been demonstrated at cell–cell contacts.^276^ The cell membrane opening can be temporary or permanent. The size of pores formed in the membrane depends on the ultrasound intensity, because of the larger cavitational shear stresses as shown in Figure 18b,c.^277^ Large carriers, such as nanoparticles or larger macromolecules, require higher ultrasound intensities compared to small molecules to permeate through the same membrane. Acoustic pressures of 190–480 kPa created pores in the size range 1 nm–4.3 μm, and for pressure amplitudes below 250 kPa the (MCF7) cells could self-heal their cell membranes.^278^ Several studies confirmed the correlation between pressure and pore size by measuring the corresponding uptake efficiency^279^ and molecular diffusion.^280^ Qiu et al.^277^ showed enhanced transfection of DNA mixed with polyethylenimine (PEI) into cells when exposed to ultrasound in the presence of microbubbles (44.7%) compared to without microbubbles (10.8%) *in vitro*. The enhanced uptake of drugs via sonoporation has been demonstrated for different kinds of substances from nanoparticles to DNA,^182,281−283^ although most of the studies have been performed *in vitro* with limited validation *in vivo*.^282^

In addition to cell membrane sonoporation, microbubble cavitation also improves drug delivery across other biological barriers, such as the blood–brain barrier (BBB) (Figure 18) and blood–spinal cord barrier.^284−286^ These barriers prevent solutes in the circulating blood from nonselectively crossing into extracellular fluid of the central nervous system where neurons reside and, hence, also prevent drug delivery to the nervous system. Similar to bubble-based cell membrane sonoporation, ultrasound-activated microbubbles can open these barriers via inertial and stable cavitation. The latter happens when the bubble size is similar to the blood capillary diameter. The bubble should be in contact with the capillary wall for efficient permeation.^287^ Depending on the microbubble size and the applied acoustic pressure, the BBB opening can be permanent or reversible. In one study BBB was shown to recover between 24 h for 1–2 μm sized bubbles driven at 0.45 MPa acoustic pressure and 5 days for 6–8 μm sized bubbles driven at 0.6 MPa acoustic pressure.^288^ Drugs can also be conjugated to the microbubbles to enhance the efficacy of delivery. Lin et al.^289^ demonstrated a gene delivery strategy via ultrasound-activated microbubbles conjugated with gene-loaded liposomes in a Parkinson’s disease mouse model. Huang et al.^290^ embedded superparamagnetic iron oxide nanoparticles on the microbubbles, which allowed magnetic guidance of the bubbles toward a specific brain region coupled to ultrasonic opening of the BBB. Beyond blood barriers, Schoellhammer et al.^291^ demonstrated that ultrasound could enhance drug delivery through the gastrointestinal tract, based on inertial cavitation.

Nanodroplets can also be used for delivery through the BBB. Chen et al.^222^ found that nanodroplets achieved a similar performance in transporting dextran across the BBB compared to microbubbles above a pressure of 0.60 MPa in a mouse model. Samples treated by nanodroplets showed no tissue damage, whereas the bubble-treated samples showed minor damage. Using nanodroplets, the BBB could be opened using pressures of 0.45–0.60 MPa (at 1.5 MHz and PRF 5 Hz).

The studies above demonstrate that ultrasonically activated microbubbles and nanodroplets are promising tools for opening biological barriers. Further research on a region-selective or disease-related BBB opening can be expected to explore conjugating the carriers with specific biomarkers and to expand testing with *in vivo* models.

### 3.5 Initiating Biological and Chemical Processes

Ultrasound can be used to deliver a chemical payload, but in some cases, it may be preferable to directly trigger a chemical reaction or a molecular change with ultrasound. Sonochemistry is a field that has long studied chemical effects caused by ultrasound.^52,292^ The traditional approach to sonochemistry utilizes high-power ultrasound to generate high pressures and temperatures that can trigger chemical processes in a bulk reactor. Recently, however, new techniques have begun to emerge that can provide control over molecular and chemical processes with much higher specificity and lower powers, making them relevant to a wider range of systems. Ultrasound can be used to generate reactive chemical species, break labile macromolecular bonds, stimulate protein complexes, or generate electric potentials to start electrochemical processes. When these processes are combined with emerging techniques such as nanocarriers, genetic engineering, or synthetic chemistry, they provide the possibility to create new kinds of systems driven directly by ultrasound.

#### 3.5.1 Chemical and Mechanobiological Triggers

##### 3.5.1.1 Sonosensitizers

**Figure 19:**
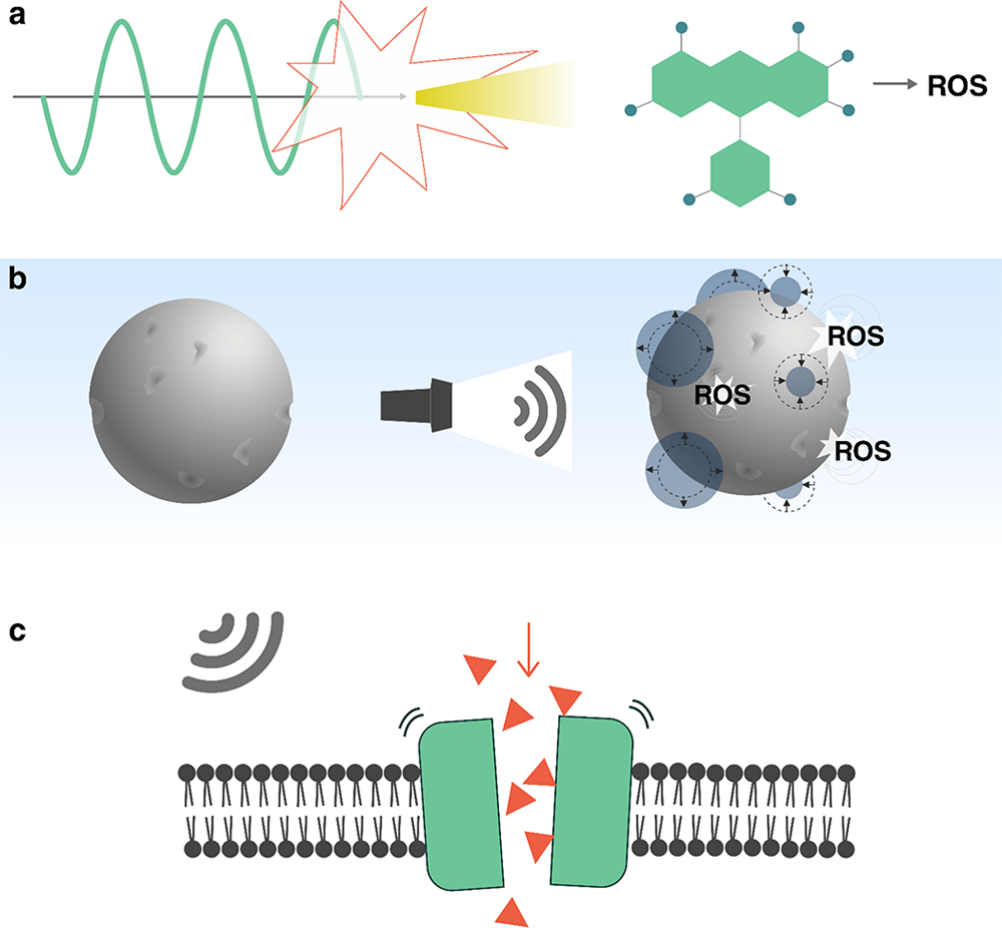
Ultrasound can be used to directly trigger or initiate (a, b) chemical and (c) biological processes. (a) Activation of molecular sonosensitizers to produce reactive oxygen species (ROS) via sonoluminiscence. (b) Production of ROS on the surface of inorganic particles via cavitation of nanobubbles. (c) Stimulation of mechanosensitive proteins using the acoustic radiation force.

Reactive oxygen species (ROS) play an important role in many chemical reactions, especially in biological systems. They are mostly known because of their deleterious effects on cells. Sonodynamic therapy (SDT) has been developing techniques that use ultrasound to control the production of ROS as a treatment for solid tumors.^201,293^ SDT is based on the use of a nontoxic sonosensitizer drug, which generates cytotoxic ROS when exposed to ultrasound in the presence of oxygen.^193,201,294^ Sonosensitizers consist of molecules^295,296^ or nanoparticles^297,298^ that are activated by low-intensity ultrasound^201^ (1 MHz, <4 W cm^–2^). The activation of molecules with ultrasound has been studied for more than 30 years,^188^ while the activation of nanoparticles dates back 10 years.^299^ More recently, improvements in SDT have been achieved by combining sonosensitive molecules with micelles^300^ or microbubbles.^199,201,294,301^ Many of the molecular sonosensitizers are also photosensitizers, which are used in photodynamic therapy where light is used to activate and generate ROS to destroy tumors.^302^ However, in contrast to photodynamic therapy, the mechanism of activation in sonodynamic therapy is not fully understood. Recently, it was proposed that the activation of the sonosensitizing molecules may proceed via sonoluminescence following the violent collapse of cavitating microbubbles in the ultrasound field (1 MHz center frequency, 3.5 W cm^–2^)^294^ (see Figure 19a). The activation mechanism for nanoparticles, however, might be different. Here it is important to distinguish between nanocarriers loaded with sonosensitizing molecules and nanoparticles used directly as sensitizers. While the loaded nanocarriers can be activated like molecular sensitizers, the nanoparticle sensitizers are activated by cavitation generated on the surface of the nanoparticle. In this case, it has been proposed that the inertial cavitation of these nanobubbles is responsible for the formation of the reactive oxygen species^303^ (see Figure 19b). Examples of nanoparticle sensitizers include superparamagnetic iron oxide nanoparticles (SPIONs), which also provide the possibility of combining ultrasound with magnetic fields for more accurate positioning. When irradiated with ultrasound (1 MHz, 1 W cm^–2^), they generate reactive oxygen species.^297^ More recently, mesoporous silica nanoparticles (MSNPs) were combined with titania and loaded with perfluorohexane to enhance cavitation when the perfluorohexane is vaporized under low-frequency ultrasound irradiation.^298^ More details on sonodynamic therapy can be found in recent reviews.^293,303,304^

##### 3.5.1.2 Mechanosensitive Proteins

Ultrasound can also be used to directly control the behavior of biological systems. The most prominent example is in the activation of mechanosensitive proteins. Ion transport between cells and neuronal activity are triggered by membrane proteins that can open to allow ion flow upon external stimulation (see Figure 19c). Channels with mechanosensitive proteins (MS channels) sense and respond to external mechanical forces, such as shear forces, or internal ones, such as osmotic pressure or membrane deformation.^305^ In the case of neurons, ultrasound has been shown to induce a mechanical stress, modulating neuronal activity. This phenomenon has been studied for some time within the context of ultrasound neural modulation (UNM),^306−311^ yet recently a new approach that combines genetic engineering with UNM has opened new possibilities to control cell function in organisms using ultrasound.^307,311,312^ This approach, known as sonogenetics, is an acoustic analogue of optogenetics and chemogenetics, where cellular function is controlled using light and chemical signals, respectively. Sonogenetics, however, has the unique potential of not requiring light or the diffusion of drugs throughout the body, since, unlike light and small molecules, ultrasound can readily penetrate deep into tissue.

Sonogenetic techniques were first demonstrated by Ibsen et al.,^312^ who showed that locomotion of the worm *Caenorhabditis elegans* could be reversed in the presence of ultrasound-driven microbubbles. This behavior was triggered by 2.25 MHz ultrasound pulses (10 ms duration) with peak negative pressures between 0 and 0.9 MPa, but only when microbubbles were present. The authors attributed this behavior to the stimulation of mechanosensitive channels by microbubble cavitation. To confirm this, they showed that by genetically engineering the worms to misexpress the protein Trp-4, which is a pore-forming subunit of a mechanotransduction channel, the worms were much less responsive to ultrasound.

Subsequent works have extended the use of ultrasound-stimulated mechanosensitive channels to other research areas.^313−326^ Besides studying behavioral changes in living organisms,^307,312,320,322−324^ sonogenetics has also been used to trigger a cellular response against tumor cells.^318,319,322,326^ For example, when T cells were engineered to express the MS channel Piezo1, ultrasound excitation in the presence of microbubbles triggered the expression of the chimeric antigen receptor (CAR), which could detect specific tumor-associated antigens.^319^

Despite MS channels being widely expressed in cells, only a few types are useful to sonogenetics. These are Piezo1, MEC-4, Trp-4, hsTRPA1, MscL, Nav, Cav, and the K2p family. The K2p family of MS channels is the only one that is inhibited by ultrasound exposure, whereas the other channel types are activated by ultrasound.^311^ The mechanism is not fully understood and remains a matter of active discussion. The most accepted potential mechanism is that ultrasound induces conformational changes on mechanosensitive (MS) ion channels, opening pores that allow the transit of ions across the membrane^307,311,327^ (see Figure 19c). Channel stimulation can be achieved either by locally induced thermal changes^323,328^ (thermosonogenetics) or by ultrasound-induced shear stresses (mechanosonogenetics),^329^ although it is not clear which one is more important. The shear stresses can be amplified by microbubble oscillations during cavitation^312,324,326^ or by fluid streaming.^307,317,325^ A major difference between the thermal and mechanical mechanisms is their activation time scale. While thermal activation takes place over seconds, mechanical activation can trigger responses within milliseconds.^307^

The growing interest in sonogenetics suggests that ongoing developments will significantly enhance the capabilities of this technique in the near future. A key goal is to unravel the precise mechanism that underlies ultrasonic neural modulation and the ultrasonic activation of MS channels. Additionally, the translation of sonogenetics to *in vivo* applications can be facilitated by developments in smart targeting methods for cavitation near the desired target, such as acoustic reporter genes (see section 3.3.3) or phase-change nanodroplets (see section 3.4.1.2). Sonogenetics will further benefit from progress in synthetic biology, which could enable applications such as control over microbe proliferation in the gut or enable control over cell growth and expression of functional payloads *in vivo*.^307^

#### 3.5.2 Mechanochemistry

While cavitation-based ultrasonics has led to sonochemistry, where chemical reactions are triggered with the generation of radicals and heat,^292^ recent work has shown that it is also possible to trigger reactions by changing the mechanical conformation of molecules. This field of research is known as mechanochemistry.^332,333^ Mechanochemical reactions are activated by bond breaking due to mechanical stimuli.^334^ Traditional mechanochemical reactions involve processes such as milling, grinding, or scratching in the solid state. In contrast, ultrasound makes it possible to trigger mechanochemical effects with ultrasound in solution.

**Figure 20:**
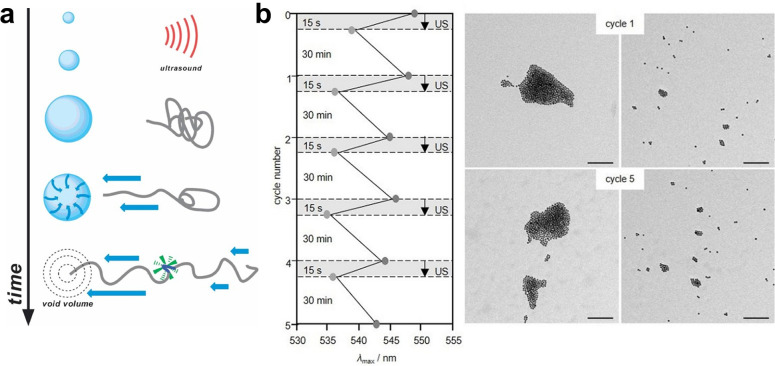
(a) Schematic illustration of ultrasound-induced mechanochemistry using cavitation. Reproduced with permission from ref (330). Copyright 2012 John Wiley and Sons. (b) Reversible assembly and disassembly of Au nanoparticle aggregates bound by a split aptamer. Disassembly took place after 15 s using 20 kHz pulses, while recovery took 30 min. Aggregate structures were tracked by using UV–vis spectroscopy (left) and TEM (right). Scale bar 100 nm. Reproduced with permission from ref (331). Copyright 2021 John Wiley and Sons.

Ultrasonic mechanochemistry makes use of molecules with an ultrasound-responsive bond, called mechanophores, that selectively break or change at predesignated sites during ultrasound exposure.^335^ The mechanophores break from shear stresses that result from unstable cavitation: bubble collapse during unstable cavitation generates large local fluid flows and intense shear stresses, which can physically stretch long molecules to break a chain (see Figure 20a). Since cavitation is required to trigger the reaction, the frequency and intensity of ultrasound used for these reactions are similar to traditional sonochemistry: the reactants are exposed for multiple hours to continuous-wave ultrasound between 20 kHz and 2 MHz,^336^ with average intensity levels of at least 3 W cm^–2^.^337^ Unlike conventional sonochemistry, however, a high level of specificity and control over the reaction is provided by the molecular design of mechanophores.

By tuning the properties of the mechanophore such as the strength and configuration of a labile bond, or the molecular weight of a chain that is activated, reactions can be triggered at precise locations and ultrasound intensities. Common mechanophore designs use a labile bond centered between large molecular chains to help break the bond via shear.^338^ Depending on the strength^339^ and configuration^330^ of the labile bonds, the mechanical force required to trigger the reaction and hence the required ultrasonic intensity are given.^340^ The molecular weight of the chain also determines the necessary ultrasonic energy: larger molecules require lower ultrasound intensity to break.^341^

The versatility and tunability of ultrasonic mechanochemistry have made it attractive for diverse applications, leading to an emergence of many new mechanophore designs and applications. By providing site-specific reactions, ultrasonic mechanochemistry has been attractive for broad applications in polymer chemistry, ranging from treating organic and inorganic compounds^342^ to self-healing components.^343^ Hu et al.^344^ developed a mechanically triggered cascade reaction that requires relatively low activation energies. This approach allowed the reaction to be triggered at room temperature and without large thermal changes to the surrounding media, which is a prerequisite for applications to temperature-sensitive biological systems. Recently, Zhou et al.^345^ demonstrated ultrasound-switchable protein activity based on a mechanophore coupled to green fluorescent protein (GFP). They applied ultrasound at 20 kHz with an intensity of 7 W cm^–2^ which stretched the protein, altering its folding stability and thereby its fluorescence brightness. Partial reversibility of the mechanism could be demonstrated. By changing the contour length of the linker structure attached to the protein (GFP-E36 to GFP-E72), they demonstrated the capability to tune the sensitivity of the protein response to ultrasound. Shi et al.^346^ showed that ultrasound could be used to simultaneously activate theranostic drug molecules and a fluorescent reporter. They designed bifunctional mechanophores composed of a disulfide bond, which was cleaved by pulsed sonication at 20 kHz with 15.84 W cm^–2^, activating the two molecules.^346^

One of the challenges in mechanochemistry is the high power levels and low frequencies (compared to clinical ultrasound), introducing potential risks for application *in vivo*. To overcome this challenge, noncovalent mechanophores have shown promise as a technique to reduce the activation energy and thus the ultrasound power. Zhao et al.^331^ used a split aptamer that interacts via hydrogen bonds and hydrophobic forces to trigger controlled release and to activate a thrombin catalyst upon ultrasound exposure. This process proceeded through the reversible disassembly of gold (Au) nanoparticles bound by the aptamer, as depicted in Figure 20b.^331^ Under focused ultrasound at 5 MHz (MI = 0.38), they achieved 75% of catalytic activity in 6 min. Although 20 kHz ultrasound with 10 W cm^–2^ can reach 50% of the activity in 15 s, these results showed reasonable effect strengths at clinical frequencies.

Ultrasonic mechanochemistry benefits from the ability of ultrasound to penetrate deeply into materials and the selective activation of different sized molecules using mechanophores. However, the technique requires an application-specific mechanophore design and high-power ultrasound to reach high reaction efficiencies.^347^ Of particular interest would be the development of universal mechanophores that can be controllably activated by low-intensity ultrasound.

#### 3.5.3 Piezoelectrochemistry

Piezoelectric materials, which generate a voltage in response to mechanical stress,^348^ are inherently responsive materials. When exposed to an ultrasonic field, piezoelectric materials can be used to trigger electrochemical reactions or to trigger a neuronal response with electric fields, and thus they have gradually emerged as materials for ultrasonically controlled chemical reactions or medical therapies.^349−351^

**Figure 21:**
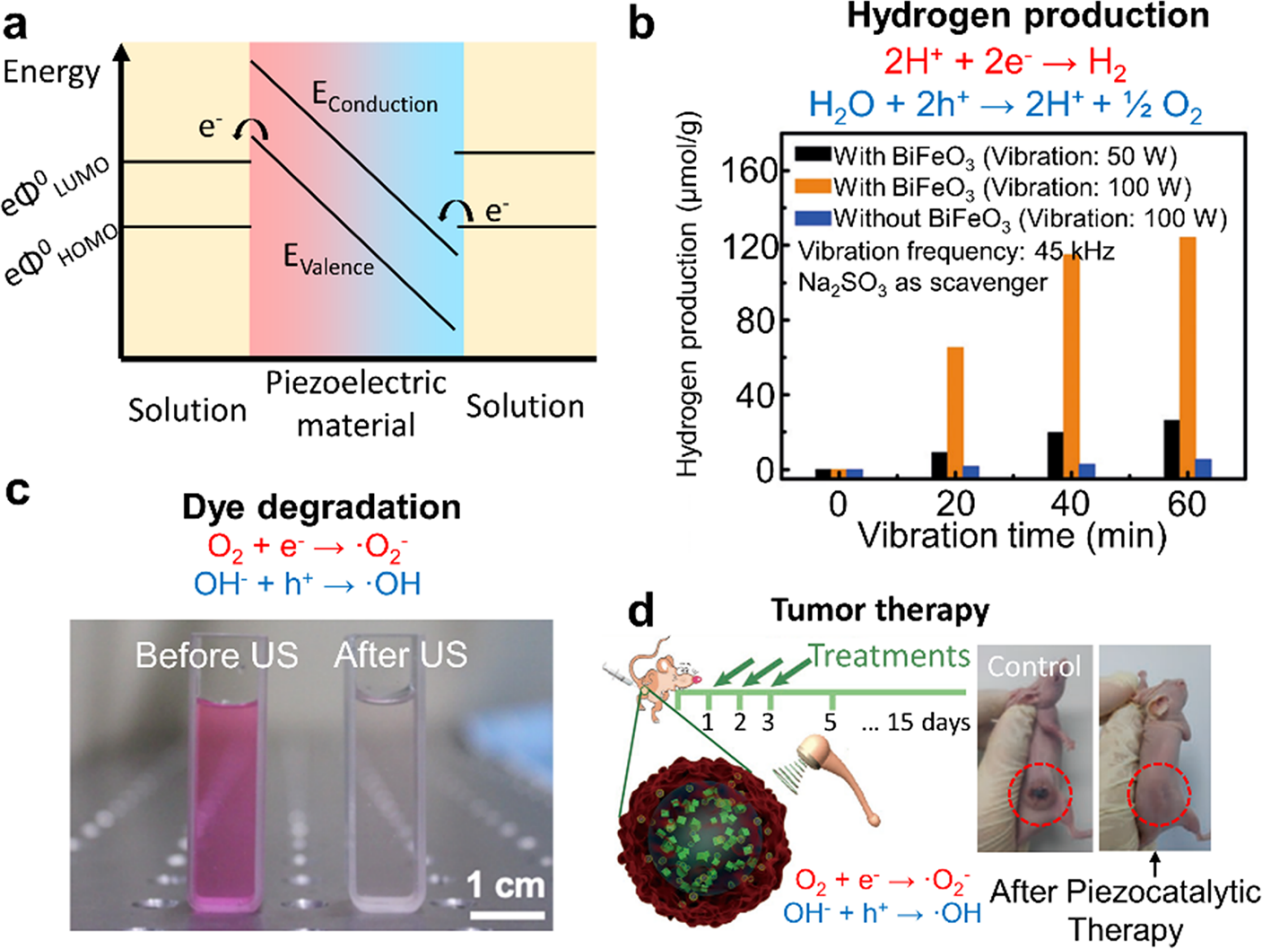
Piezoelectric materials for ultrasound-actuated electrochemical reactions. (a) Schematic for a piezoelectricity-induced chemical reaction: strain in the piezoelectric material generates electrical charges that change the energy state across the material, facilitating electron transfer between the material and the surrounding solution. Adapted with permission from ref (351). Copyright 2015 Elsevier. (b) Result of ultrasound-induced hydrogen production and its correspondence to the presence of piezoelectric material and the applied acoustic power. Adapted with permission from ref (352). Copyright 2019 John Wiley and Sons. (c) Photograph of ultrasound-actuated dye degradation and the experimental evaluation before and after the ultrasonic excitation. Adapted from ref (353). Copyright 2019 American Chemical Society. (d) Ultrasound-triggered generation of reactive oxygen species (ROS) and its application in tumor therapy. (insets) Digital photos of 4T1-tumor-bearing mice after treatment with ultrasound-activated BaTiO_3_ nanoparticles (right) and the control group without treatment (left). Adapted with permission from ref (354). Copyright 2020 John Wiley and Sons.

When an insulating piezoelectric particle suspended in a liquid is exposed to an ultrasonic field, the ultrasonic vibrations will induce an oscillating electric polarization due to the piezoelectric effect. The associated electric field causes an energy shift of the valence and conduction bands throughout the piezoelectric material. When the energy shift is comparable to the difference in energies of the lowest unoccupied molecular orbital (LUMO) and highest occupied molecular orbital (HOMO) of a molecule in solution, eletctron transfer between the piezoelectric material and the species in the solution becomes possible, thus triggering a chemical reaction (Figure 21a).

Piezoelectric particles have been developed for multiple applications of ultrasound-driven chemistry. One of the early applications was for hydrogen production via water splitting^352,355,356^ (Figure 21b) . Different piezoelectric particles have been used for this, including ZnO nanorods^356^ and BiFeO_3_ nanosheets.^352^ For example, You et al.^352^ achieved a 124.1 μmol g^–1^ hydrogen production rate with BiFeO_3_ nanosheets using 100 W of 45 kHz ultrasound applied for 1 h. In addition to hydrogen production, piezoelectric materials have also been used for dye degradation during wastewater treatment (Figure 21c).^353,357^ Wu et al.^357^ reported degradation of rhodamine B using ultrasound-actuated few-layer MoS_2_ nanoflowers. Compared to nonpiezoelectric control samples that used multilayer MoS_2_ or TiO_2_, the few-layer MoS_2_ particles with strong piezoelectricity showed a significantly faster degradation rate under the same ultrasonic conditions. The nanosized piezoelectric particles disperse well in wastewater; however, they are difficult to remove after the water treatment. To solve this issue, Qian et al.^353^ developed a composite ultrasound-responsive foam by mixing piezoelectric BaTiO_3_ microparticles with an elastomer, polydimethylsiloxane (PDMS). The porous foam can degrade the dye in solution actuated by ultrasound and can be more easily collected after the treatment.

Ultrasound-triggered piezoelectrochemical reactions can also be used to generate cytotoxic radicals, such as reactive oxygen species (ROS) for tumor therapy (Figure 21d). Zhu et al.^354^ reported ultrasound-triggered piezoelectric BaTiO_3_ nanoparticles for generating ROS for targeted tumor treatment. The piezoelectric nanoparticles were encapsulated in a hydrogel, and the composite was injected near the tumor. When exposed to ultrasound, the piezoelectrochemical reaction generated cytotoxic hydroxyl and superoxide radicals in the targeted region. *In vivo* experiments on mice verified that the therapeutic process was both effective and biocompatible. In addition to naturally occurring piezoelectric materials, Wang et al.^358^ also demonstrated that the inert poly(tetrafluoroethylene) can be ultrasonically activated to exhibit piezoelectricity and then applied for ROS generation.

**Figure 22:**
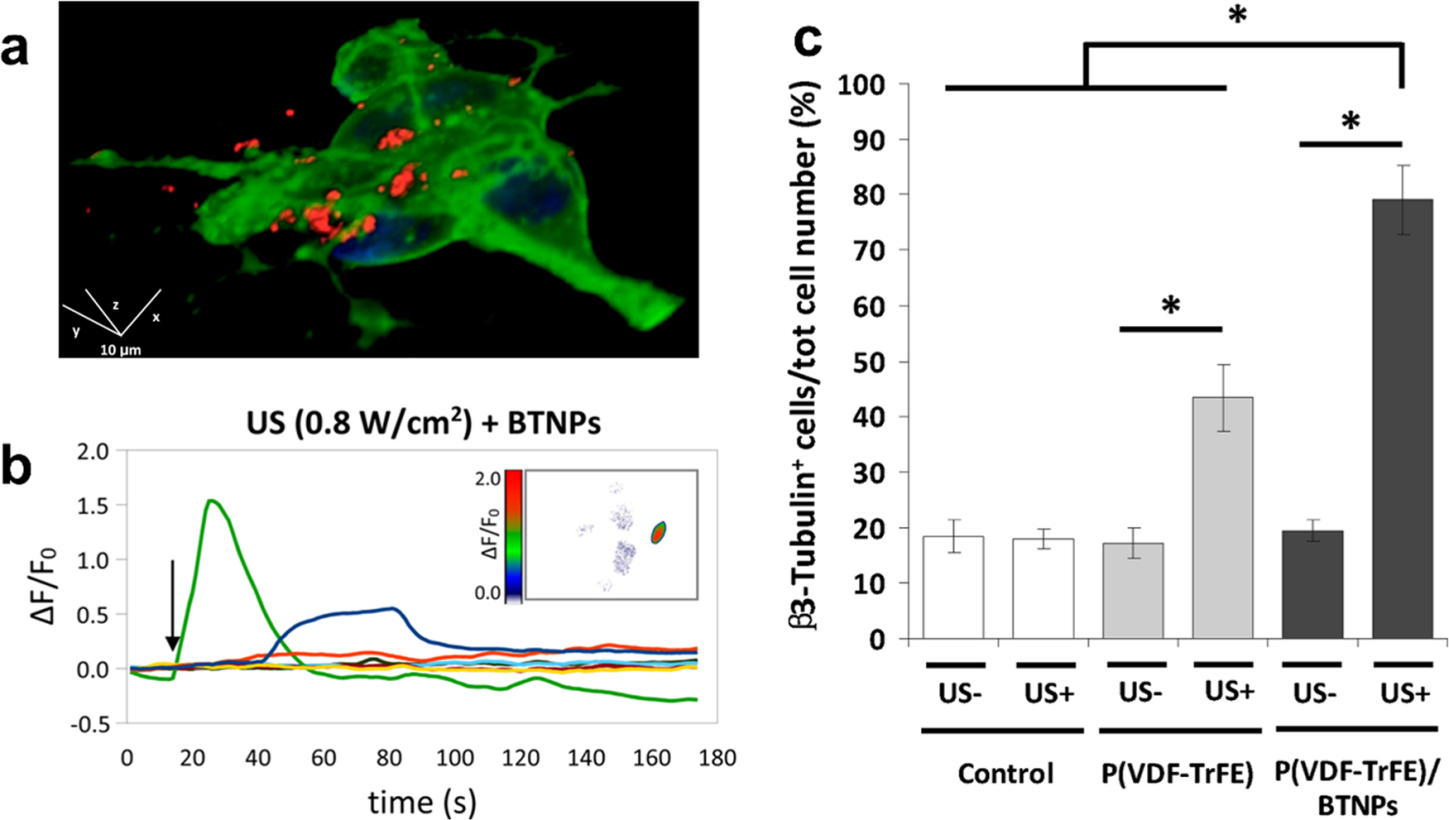
Piezoelectric materials for ultrasound-triggered cell stimulation. (a) Confocal fluorescence microscopy of piezoelectric BaTiO_3_ nanoparticles (red) attached to neuronal plasma membranes (green). (b) High-amplitude calcium ion transients (green curve) were observed with respect to an ultrasound (US) trigger (time point indicated by arrow) applied to the BaTiO_3_ nanoparticles (BTNPs). Panels a and b reproduced from ref (359). Copyright 2015 American Chemical Society. (c) Percentages of β3-tubulin positive cells (biomarker indicating differentiation) under different conditions verify the enhanced differentiation under synergistic action of ultrasound and piezoelectric nanoparticles. US–/+, ultrasound off/on; Control, without nanoparticles; P(VDF–TrFE), poly(vinylidene fluoride–trifluoroethylene); BTNPs, BaTiO_3_ nanoparticles. Reproduced with permission from ref (360). Copyright 2016 John Wiley and Sons.

In addition to inducing electrochemical reactions, the electrical charges generated by piezoelectric particles in an acoustic field can also be used for cell stimulation. Marino et al.^359^ demonstrated neural stimulation by ultrasonically activated piezoelectric BaTiO_3_ nanoparticles dispersed in cell culture media. Cellular responses such as calcium transients through the cell membrane were observed by fluorescence imaging of the ion dynamics after treatment by 1 MHz ultrasound and piezoelectric nanoparticles (Figure 22a,b). In another test of piezoelectric nanoparticles for cell stimulation,^361^ the electrophysiological response of a cell culture was measured with a microelectrode array patterned on the cell culture. The measurement showed that the combination of piezoelectric nanoparticles and ultrasound triggering could significantly increase neuronal activity (quantified by mean firing rate of the network of neurons). Finally, Marino et al.^362^ showed that piezoelectric BaTiO_3_ nanoparticles could be embedded into a 3D-printed microstructure for spatially resolved ultrasound stimulation of cells. Human sarcoma osteogenic cells were cultured on the microstructures, showing enhanced osteogenic differentiation (higher deposition of hydroxyapatite nodules) after ultrasound exposure compared to a control group without ultrasound exposure. Using a similar principle, enhanced cell differentiation in human neuroblastoma cells was observed on an ultrasound-activated piezoelectric film, which was made of poly(vinylidene fluoride–trifluoroethylene) and BaTiO_3_ nanoparticles^360^ (see Figure 22c).

### 3.6 Actuation and Locomotion

Ultrasound can be used to actuate smart systems. It can induce fluid flows or propel objects such as cells or micromotors through a liquid. Its biocompatibility and good transmission through tissue mean that ultrasound can induce motion in hard-to-access regions, which makes it promising for biomedical applications of smart devices.

#### 3.6.1 Controlling Fluid Flow

Ultrasound can induce fluid streaming either in the path of the propagating waves or at oscillating fluid boundaries (as discussed in section 2.3.2). This has led to a number of applications of acoustic streaming in biology and for lab-on-a-chip devices which are discussed in this section. Early work on integrated devices focused on the use of surface acoustic wave (SAW) streaming effects, which were discovered by Shiokawa et al.^363−365^ and later developed by Wixforth et al.^366−368^ SAW techniques for fluid pumping and manipulation have been covered in recent comprehensive reviews on SAW microfluidics.^6,14,369^ Here, we focus primarily on acoustic-driven systems that utilize oscillating bubbles or solid structures to control the fluid flow or to manipulate micrometer to millimeter sized objects.

The role of streaming in controlling an object is governed by the object’s size. Bubble oscillations in an acoustic field produce an attractive radiation force on micrometer sized objects in the bubble’s near field. At the same time, the oscillating bubble generates streaming flows, which can exert a force on nearby objects via viscous drag in the fluid.^370^ Whereas the radiation force scales with the cube of the particle radius (*F*_R_ ∝ *R*^3^), the streaming-induced force scales linearly with the particle radius (*F*_S_ ∝ *R*).^371^ For example, for bubbles resonant on the order of hundreds of kilohertz, streaming is more effective for objects smaller than 10 μm whereas for larger objects the ARF becomes dominant.^372,373^ For objects tens of micrometers in size, acoustic forces in the range 1–10 nN can be generated by resonant microbubbles oscillating at tens of kilohertz, and streaming velocities at the bubble surface can reach values of 5–20 mm s^–1^ on resonance.^373,374^

**Figure 23:**
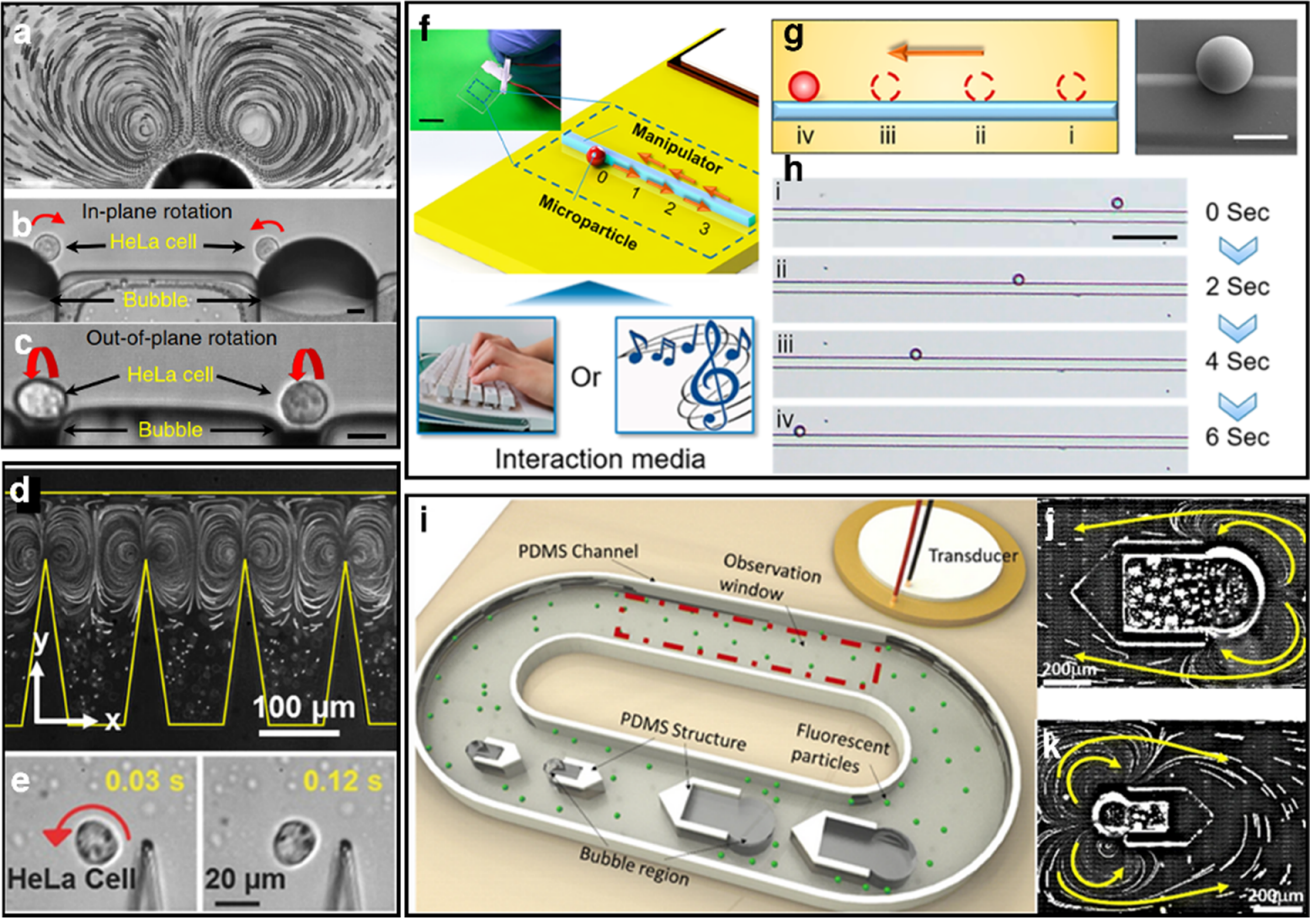
(a) Experimental demonstration of acoustic streaming during microbubble oscillation at 24 kHz (scale bar 30 μm). In-plane (b) and out-of-plane (c) rotation of a HeLa cell, driven by an oscillating microbubble at a constant frequency (scale bar 10 μm). Panels a–c reproduced from ref (375). CC BY 4.0. (d) Acoustic streaming vortices generated by oscillating sharp-edge structures rotate (e) a HeLa cell. Panels d and e adapted with permission from ref (376). Copyright 2016 John Wiley and Sons. (f) Structural design and configuration of the acoustics-based human microrobot interface platform (inset scale bar 10 mm) which includes a piezoelectric transducer (white), a glass substrate (yellow), a line-shaped micromanipulator (light blue), and a micro particle transported to the destinations (0, 1, 2, 3). (g) Schematic of particle transportation (right, scanning electron microscopic image of the microparticle, scale bar 10 μm)) and corresponding microscopic images (h) with a time lapse of 2 s (scale bar 50 μm). Panels f–h reproduced from ref (377). Copyright 2019 American Chemical Society. (i) Configuration of the acoustically driven bidirectional micropump. (j, k) Acoustic microstreaming flow induced by different sized bubbles. Panels i–k reproduced with permission from ref (378). Copyright 2020 Springer-Verlag GmbH.

Acoustic streaming flows can be used for different trapping and manipulation tasks at microscales. Micro objects can be trapped by localized streaming vortices, which are visualized in Figure 23a. For instance, Ahmed et al.^375^ captured and manipulated the nematode *C. elegans* in solution using arrays of acoustic-driven oscillating microbubbles in a microfluidic device. Acoustically-excited 250 μm bubbles at the hydrophobic walls of the device were used to trap the nematode via the acoustic radiation force, and the streaming flows caused its rotation. Further, Ahmed et al.^375^ demonstrated the in-plane and out-of-plane rotation of HeLa cells (Figure 23b,c) by simultaneous coupling of radiation force and acoustic microstreaming vortices for trapping and manipulation, respectively. Similarly, acoustic-streaming-based manipulation has been used to control other biosamples such as *C. elegans*,^379^ zebra fish,^380^ and pollen.^381^ Beyond simple manipulation, microstreaming flows generate shear stresses that can deform or even rupture small soft objects.^40^

Apart from bubbles, streaming flows can also be generated by oscillating microstructures. Hayakawa et al.^382^ used three (200 μm) micropillars arranged in a triangular configuration to generate circulating flows to transport and rotate mouse oocytes. Ozcelik et al.^376^ presented an on-chip acoustofluidic device that achieved rotation of single HeLa cells using steady streaming vortices (Figure 23d,e). Maximum flow rates on the order of 5 mm s^–1^ were predicted for the device, and rotation rates up to 60° s^–1^ were observed. These flows were generated by resonant oscillations of the micropillars at 5 kHz with an amplitude between 0.5 and 5 μm. The streaming effects induced by oscillating microstructures have also been used to transport particles.^371,383^ Lu et al.^377^ presented a user-controlled platform to manipulate microparticles with locally enhanced acoustic microstreaming along a fixed pathway (Figure 23f–h). By combining oscillating microstructures with viscoelastic fluid media, Zhou et al.^384^ showed that the streaming induced by vibrating micropillars can even be used to concentrate submicrometer particles.

Ultrasound-generated streaming flows can also be used to develop efficient pumps for lab-on-a-chip devices. Ryu et al.^385^ realized a microfluidic pump based on millimeter sized bubbles oscillating in water and achieved a flow rate of 0.6 μL s^–1^. Similarly, Gao et al.^378^ presented a bidirectional micropump by arranging different sized resonant microbubbles in a channel (Figure 23i). Because of the distinct resonant frequencies of the different bubbles, the flow direction could be controlled by switching the excitation frequency. The streaming flow pattern inside the channel around the different sized bubbles are shown in Figure 23j,k. Micromixers for lab-on-chip devices have been realized with microbubbles.^386−390^ Vibrating sharp-edge microstructures have also been utilized to build micropumps and micromixers in microfluidics. Huang et al.^391^ designed an acoustofluidic micromixer based on oscillating 250 μm sharp-edge structures excited at 4.5 kHz. With an identical working principle, Huang et al.^392^ created a programmable microfluidic pump by orienting an array of 20 sharp-edge structures 30° relative to the channel wall.

Viscous streaming flows have recently emerged as a powerful tool to generate flows in featureless small channels. For example, Huang et al.^393^ demonstrated that 100 MHz surface acoustic waves (SAW) could be used to drive viscous streaming flows within a lithium (Li) battery electrolyte. The induced flows minimized Li dendrite formation, increasing charging performance. By incorporating the SAW, they showed that it is possible to use lithium metal as an anode in a rechargeable battery for the first time.^393^ In an even more recent study, Zhang et al.^394^ identified a new nonlinear mechanism for SAW-driven streaming flows in nanoscale channels, which produced flow rates up to 6 mm s^–1^ in a 150-nm-tall nanoslit. These observed flow rates are more than 10 times higher, and the flow pressures more than 10^3^ times higher, than those predicted by any other mechanism.^394^ This acoustogeometric streaming mechanism represents a unique new direction for applications requiring fast flows in nanoscale channels.

Ultrasonic streaming techniques present numerous advantages,^395^ including noncontact operation and suitability to manipulate cells and microorganisms.^379^ The devices are inherently compact, and operation at higher frequencies presents the opportunity for more compact integrated devices using SAW.^6,369^ These active acoustic systems can be incorporated into smart devices for noncontact fluid pumping, handling, and manipulation of objects.

#### 3.6.2 Actuating Individual Particles and Swimmers

The emerging field of micro- and nanorobotics is especially receptive to smart materials because the small size of individual components prohibits the classic modular approach in macroscale robotics with onboard computation and memory. A review on smart materials for microscale robotics has been written by Soto et al.^396^ Ideally, the responsive behavior is encoded in the structure of the microrobot and can be controlled by an external field. Here, we focus on recent developments using acoustically initiated responses for propulsion and actuation in microsystems.

**Figure 24:**
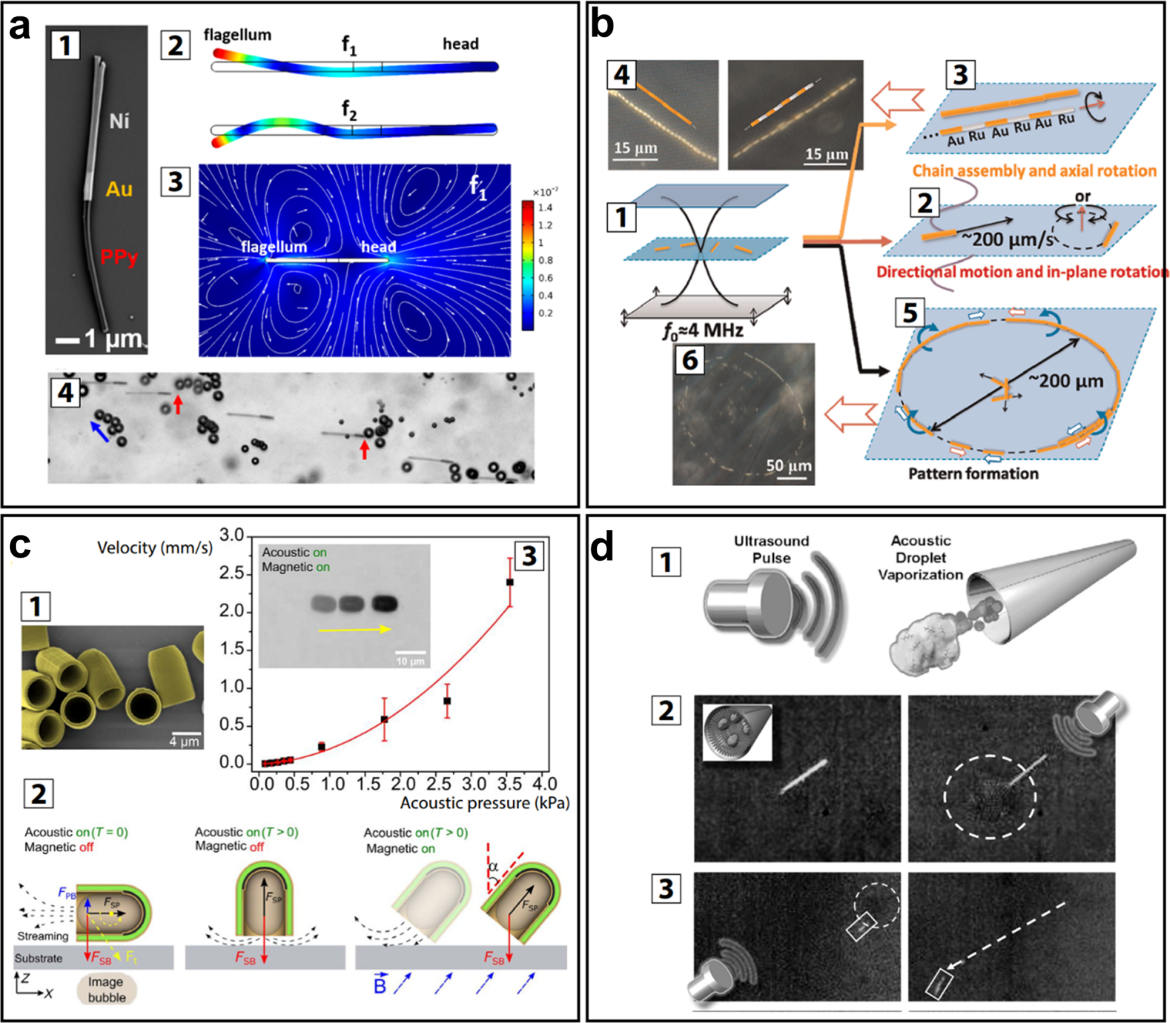
Acoustic microswimmer concepts. (a) Forced body oscillations. Adapted from ref (397). Copyright 2016 American Chemical Society. (b) Asymmetric steady streaming. Reproduced from ref (398). Copyright 2012 American Chemical Society. (c) Bubble streaming. Adapted from ref (399). CC BY-NC 4.0. (d) Acoustic droplet vaporization. Adapted with permission from ref (400). Copyright 2012 John Wiley and Sons. Detailed descriptions of subpanels are in the main text.

A major focus in microrobotic systems is controlled propulsion to a target area.^401^ Ideally, untethered actuation can be achieved in a variety of media, including biological ones, enabling minimally invasive medical interventions. A review by Nelson et al.^402^ presents the state of the art in medical microrobots and discusses potential applications. Several concepts for acoustically induced propulsion of microrobots have been proposed (Figure 24), based on forced body shape changes,^397,403^ asymmetric steady streaming,^398,404−410^ bubble streaming,^370,399,411−413,413−419^ or nonreversible jetting caused via rapid vaporization of a fuel.^400^ Note that some of the cited examples are not biocompatible, e.g., through incorporation of nickel or the addition of a catalytic motor based on decomposition of hydrogen peroxide, which prohibits use *in vivo*. However, the four acoustic actuation mechanisms described above are generally biocompatible and thus provide an advantage over other currently researched actuation concepts such as catalytic nanomotors.

An artificial swimmer can for example use structural resonances of its body to propel itself forward (Figure 24a). Ahmed et al.^397^ presented such a swimmer fabricated by a template electrodeposition technique, which consists of a bimetallic head and a polypyrrole tail (Figure 24a1). The swimmer moved at velocities of up to 50 μm s^–1^ when driven at its fundamental resonance frequency of 91.5 kHz. Parts a2 and a3 of Figure 24 show the first bending modes and the flow developed around the swimmer at the first resonance, respectively. The swimmer motion relative to other tracer particles can be seen in Figure 24a4.

Asymmetric microswimmer design can also lead to fast self-propulsion in an acoustic field. Wang et al.^398^ investigated the behavior of metallic microrods in an ultrasound field, shown in Figure 24b. The microrods (2 μm length and 330 nm diameter) were first pushed toward the nodal plane of a standing acoustic wave (*f* = 3.7 MHz) inside a closed chamber (Figure 24b1), where they displayed linear motion and rotation (Figure 24b2). The rods’ fabrication process (template electrodeposition) resulted in asymmetric shapes with one end being flat (head) and the other end concave shaped (tail). The rods were made from a varying number of metals and always moved head first with speeds up to 200 μm s^–1^ (or 100 body lengths per second). This is surprising, since their size is much smaller than the corresponding acoustic wavelength (400 μm). The high speeds are thought to arise from asymmetric steady streaming, where an asymmetric particle oscillates in a uniform field (true in the pressure node for a particle with size *a* ≪ λ) and thereby induces a net flow.^406^ Many rods interacted and formed chains connected from head to tail, which then moved collectively in lines (Figure 24b3,b4) or circles (Figure 24b5,b6) around the nodal region.

Bubbles embedded in a microswimmer can also be used for propulsion. Figure 24c1 shows a bubble-based microswimmer described by Ren et al.^399^Figure 24c2 depicts the working principle, which is based on the primary and secondary Bjerknes forces *F*_PB_ and *F*_SB_ as well as the force caused by the bubble’s cavitation microstreaming, *F*_SP_. The authors found *F*_PB_ ∝ *ϵr*^3^*P*_ac_*f*/*c*_L_ and *F*_SP_ ∝ ϵ^2^ρ_L_*r*^4^*f*^2^, where ϵ is the amplitude of the bubble’s surface vibration, *r* is the bubble radius, *P*_ac_ is the acoustic pressure, *f* is the acoustic frequency, and *c*_L_ and ρ_L_ are the speed of sound and the density of water, respectively. For a 4 μm bubble driven at 1.33 MHz the authors find the streaming propulsive force to be 10 times stronger than the primary radiation force. However, the secondary radiation force attracts the bubble to a surface with *F*_SB_ ∝ 1/*d*^2^, where *d* is the distance between the bubble and the surface. Close to a wall this causes the swimmer to approach it, rotate, and eventually point in the normal direction—which stalls its forward movement. To generate forward thrust, the authors aligned the nickel (Ni)-coated capsules with a magnetic field as shown in the right panel of Figure 24c2. The velocity could be set by changing the magnetic field direction. Even at a relatively low acoustic pressure of 4 kPa, the microswimmer was propelled at a speed of up to 2.5 mm s^–1^ (350 body lengths s^–1^) (Figure 24c3).

Acoustic droplet vaporization (see section 3.4.1) is another technique that has been used to propel a nanorocket (Figure 24d). Kagan et al.^400^ fabricated nanorockets in sizes ranging from 8 to 40 μm by stressed metallic (Au, Ni) thin film release or template electrodeposition. A crucial fabrication step was to functionalize the inner gold surface with cysteamine, which allowed a drop of perfluorohexane (PFH) emulsion to be trapped inside the cavity. Due to the slightly conical shape of the tube, the PFH was expelled via the larger opening once it had been vaporized by an acoustic pulse. A magnetic Ni layer permitted magnetic steering. When actuated, the rockets traveled at speeds over 6 m s^–1^. The authors demonstrated penetration 200 μm into tissue samples from a lamb kidney using pressure pulses ranging from 1.6 MPa over 44 μs to 3.8 MPa over 4.4 μs, without observing cavitation effects.

Bubble-based streaming actuators can be scaled up to generate larger forces or manipulate larger structures. Qiu et al.^417^ reported actuators that use large arrays of microbubbles. When remotely powered with ultrasound, the surface generated a propulsive force on the order of 1 mN. By combining multiple actuators with varying resonance frequencies between 20 and 100 kHz, a robotic arm could be actuated in multiple directions, where motion along each degree of freedom could be selected via the corresponding driving (resonance) frequency. The sound amplitudes required to operate the device were below 200 kPa.

Microrobots can themselves be seen as building blocks for smart materials, especially when they exhibit emergent collective behaviors.^144^ Ultrasound may induce collective effects directly through aggregation phenomena via the primary and secondary radiation forces.^420,421^ In addition, a combination of many interacting forces, such as magnetism and optical forces, can give rise to more complex behaviors.^422,423^ For example, Xu et al. demonstrated how the acoustic radiation force is about 8 times larger than chemical forces, but their interplay can be used to dynamically change the configuration of the micromotors.^424^

## 4 Conclusion and Outlook

Recent developments demonstrate the growing capabilities that ultrasound can provide to realize responsive systems. We have reviewed how ultrasound has been applied in diverse contexts to support smart capabilities. These can be grouped into six categories: directed assembly, modulation of material properties, sensing, payload transport and delivery, triggering of biochemical processes, and actuation and locomotion. These capabilities are enabled by several ultrasound mechanisms: cavitation, acoustic streaming, ultrasound-induced vibration, acoustic scattering, and acoustic radiation forces. By combining different mechanisms with creative system designs, unique capabilities have been realized for applications ranging from optical communication and imaging, to water treatment, to drug delivery and *in vivo* sensing of gene expression.

Moving forward, we expect to see a growth of applications for ultrasound-enhanced materials and microsystems. Emerging areas will also impose new constraints and demand more capabilities of their materials than the current systems do. We believe the demands of next-generation applications can be met in the short term by developments in three directions.

First, the ultrasonic capabilities described here can be applied in nontraditional ways when translated to new domains. Most current research is oriented toward biomedical applications, and this has been the main driver for many technological advances. However, other fields such as agriculture, construction, or biosensing will also benefit from the development of smart ultrasound systems. For example, agriculture can benefit from smart delivery to promote plant growth under stressful conditions.^253^ In construction, ultrasound-induced self-healing of cements^425^ could support longer-lived smart cities. New bioimaging technologies may benefit from ultrasound-switchable fluorescence (USF).^426,427^ New insights and capabilities will likely emerge from such cross-disciplinary application of ultrasound-mediated effects.

Second, individual ultrasound-responsive systems should be pushed to provide multiple functionalities in constrained applications. It has already been shown, for instance, that gas vesicles can be used for both biomedical sensing and therapy. Similarly, micro- or nanobubbles can be used for both transport of reactants and initiation of chemical processes. Combining multiple responses into a single system can allow greater functionality in applications with many constraints.

Finally, the ultrasound-induced effects described above should be combined also with other phenomena (e.g., optical effects) to achieve additional capabilities and more precise, nuanced control over their activation. Combinations of microbubbles and magnetic particles were shown to increase the localization efficiency for barrier opening and drug delivery. Alternately, combining optical excitation with ultrasonic excitation of gold nanoparticles lowered the cavitation threshold needed for payload delivery. Such combinations can improve the specificity of targeting with ultrasound-driven systems, reducing unwanted collateral effects.

In the longer term, fundamental questions will need to be answered alongside application-driven development. Some arising topics of interest that still require significant work to address include the following:

• How can ultrasound fields be shaped with high complexity and temporal tunability? Current techniques to shape sound fields rely on either transducer arrays, which provide good temporal control but poor spatial control, or holograms, which provide good spatial control but poor temporal control over the pressure field. What kinds of developments in materials,^428^ acoustic metamaterials,^429,430^ or other hardware will allow for high-resolution, dynamic sound modulation?^75^

• How can dynamic ultrasound effects and materials be used for real-time feedback (e.g., haptic^431^) and control in human-interfaced systems?

• How can ultrasonic subsystems be integrated with computerized microrobotic devices? A new generation of remotely programmable microrobots with built-in computers can perform tasks at cellular length scales.^432^ How can ultrasonic subsystems be integrated with such robots to enhance their propulsion, sensing, or manipulation capabilities?

• How can we confine ultrasound to smaller regions with higher intensity, for more localized control of ultrasound-dependent processes? Currently, it is easier to focus sound at higher frequencies where the sound attenuates more quickly. Besides bubbles, what kinds of systems can be used to localize acoustic energy the same way that fiber optics and nanoresonators do for optics? How can similar techniques be applied for higher-resolution ultrasound sensing?^433^

• Is it possible to trigger chemical and biological processes with low-intensity ultrasound, even in the absence of cavitation?^434^ What are the mechanisms that enable this, and how can they be adapted for use in different systems? Some approaches based on ultrasonic heating and streaming have been shown to have an effect on chemical processes,^435,436^ but more research is needed to identify and understand molecular-level effects.

• Can ultrasound be used to activate biological processes and trigger localized neuronal action? Exploration of the mechanisms for such processes is needed at a fundamental level to ultimately provide control in applications such as neuromodulation and real-time biocontrol.

• How can innovations in materials and microfabrication^437^ help ultrasound to interact with other physical fields or phenomena that are currently difficult to couple? Interesting examples include stronger coupling of ultrasound with light, mechanical properties of materials,^143^ and even quantum bits.^438,439^

Addressing these questions may provide insights and lead to innovations that can extend the applicability and capabilities of ultrasound-responsive systems. The traditional benefits of ultrasound make it a valuable tool for a broad range of applications. It can be used for imaging, to transfer energy effectively through complex and opaque systems, and to localize that energy into small regions in space. Ultrasound can couple to systems across a wide range of length and time scales, providing both nondestructive and destructive effects. As the demand for multifunctional, responsive smart systems and materials increases, emerging systems can leverage the benefits of ultrasound, adapting ultrasound-driven phenomena to support and enable diverse smart functionalities.
